# Changing pattern of exposure to polycyclic aromatic hydrocarbons over time in the Central European population

**DOI:** 10.1038/s41370-025-00793-z

**Published:** 2025-07-17

**Authors:** Soňa Smetanová, Akrem Jbebli, Jiří Kohoutek, Vladimíra Puklová, Milena Černá, Andrea Krsková, Martin Zvonař, Zdenko Reguli, Lenka Andrýsková, Pavel Piler, Petra Přibylová, Jana Klánová, Elliott J. Price, Klára Komprdová

**Affiliations:** 1 RECETOX, Faculty of Science, Masaryk University, Kotlarska 2, Brno, Czech Republic; 2 National Institute of Public Health, Šrobárova 48, Prague, 10 100 42 Czech Republic; 3 Charles University, Third Faculty of Medicine, Prague, Czech Republic; 4 Faculty of Sports Studies, Masaryk University, Kamenice 753/5, Brno, Czech Republic

**Keywords:** Polycyclic aromatic hydrocarbons, Legislation, Chemical exposure, Biomonitoring

## Abstract

**Background:**

Temporal trends of chemicals in the population are key to identifying changing sources of chemicals and determining the effectiveness of various legislative measures.

**Objective:**

The present study focused on time comparisons to explore a possible decrease in PAH metabolite levels in the Czech population. Legislative measures occurred between sampling periods, including restricting smoking and the Air Protection Act.

**Methods:**

Ten metabolites of PAHs were measured in urine samples collected in 2011–2012 from mothers and children from DEMOCOPHES-CZ study (*N* = 235) and in 2019–2020 from children, teenagers, and young adults from CELSPAC studies (*N* = 809). Multivariate linear regression, Kruskal-Wallis ANOVA, and Mann-Whitney test (MW) were used to investigate differences in OH-PAHs between periods, age categories, and exposure determinants.

**Results:**

Median concentrations significantly decreased between 2011-2020 by 30–35% for 1-OH-NAP, 2-and 3-OH-FLUO, 85% for 1-OH-PHE, and 44% for 2/3-OH-PHE, while 2-OH-NAP increased by 29% in non-smoking adults. In children, median concentrations of all metabolites decreased by 10–51%, with 2-OH-NAP rising by 49%. Smokers showed the largest differences, with significant decreases of 46–59% in the median concentrations of 2-OH-NAP, 2/3-OH-PHE, 9-OH-PHE, and 1-OH-PYR, and 76–91% in OH-FLUOs, 1-OH-NAP, and 1-OH-PHE. Fish and offal consumption, season, locality, and type of cooking were significant factors associated with levels of OH-PAHs, explaining 4–9% of the variability. Smoking was the main contributor in 2011, explaining up to 45% variability; no difference was found between smokers and non-smokers in 2019. New reference values of OH-PAHs in urine were calculated for the Czech population.

**Impact:**

- This study analyses the temporal trends of OH-PAHs in the population in the context of introduced legislative measures. In addition, it examines OH-PAH exposure in children, adolescents, and young adults in relation to lifestyle factors and establishes new reference values for polycyclic aromatic hydrocarbons that are important for public health decision-making. Biomonitoring over time is an essential tool for establishing new measures to protect public health.

**Figure:**
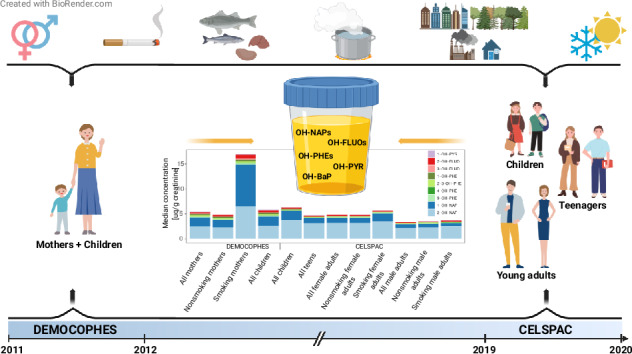
Created in BioRender. Komprdova, K. (2025) https://BioRender.com/u10q831.

## Introduction

Polycyclic aromatic hydrocarbons (PAHs) are persistent organic pollutants (POPs) widespread in the environment and recognised as hazardous to human health and the environment. The main source of environmental contamination is incomplete combustion of carbon-rich materials during energy production and industrial processes [1]. Other major anthropogenic sources of PAH pollution are vehicular emissions, agricultural practices, and household heating [2–4].

Human exposure to PAHs is via inhalation, ingestion, or digestion of contaminated food and dermal absorption [5]. The diet is considered the greatest source of exposure for non-smokers [6, 7], though exposure via inhalation has been proposed to have greater toxicological risk [8]. Human exposure to PAHs has been associated with higher all-cause mortality [9], heightened risk of developing cancers and cardiovascular conditions [10], and reproductive and developmental issues [11, 12]. In particular, children have been indicated to be at greater risk of PAH exposure and potentially more susceptible to adverse health effects compared to adults [13].

Upon entering the human body, PAHs are converted to more polar metabolites and preferentially excreted in urine, yet, higher mass analytes (>500 Da) are also removed in faeces [14]. Biotransformation predominantly occurs via Cytochrome P450 enzymes (CYP) of the liver [15] and hydroxylated PAHs (OH-PAHs) are the most abundant PAH metabolites in the human body. Consequently, the measurement of urinary OH-PAHs is widely applied for population biomonitoring of PAH exposure [14]. Biomonitoring studies have commonly focused on the measurement of urinary pyren-1-ol (1-OH-PYR) as an indicator of overall PAH exposure [16]. There are several studies investigating non-occupational OH-PAH levels in children and adolescents of the general population [17–23]. The study of a wider array of biomarkers is advocated [19], and the determination of specific urinary biomarkers and the establishment of their threshold levels for health risk assessment are subjects of ongoing investigation [7].

Globally, international/national biomonitoring programs strive to define reference levels and mitigate exposure via legislative measures. In Europe, initiatives like DEMOCOPHES (Demonstration of a study to Coordinate and Perform Human Biomonitoring on a European Scale) and HBM4EU (European Human Biomonitoring Initiative) prioritised PAHs for biomonitoring, advocating for policy interventions [24, 25].

The levels of OH-PAH exposure in Europe were evaluated over time for three time periods: 2000–2010 based on literature data, biobank samples from the DEMOCOPHES 2011–2012 project, and the HBM4EU 2014–2021 aligned studies, but no clear decreasing/increasing trend in urinary OH-PAHs were observed [26]. After the 2005 regulation setting maximum levels for PAHs in foodstuffs (Commission Regulation (EC) No. 1881/2006 of 19 December 2006), a decreasing trend in urinary concentrations of PAH metabolites was observed, followed by an increase that peaked around 2017, after which levels declined again. This concave downward trend was consistent across most PAH metabolites. Whilst aggregated results may help to identify longer-term temporal trends in the European population, trends in OH-PAHs may differ by region due to individual countries varying in culture, diet, and often regulations [26]. National studies that include questionnaire data on lifestyle, socio-economic status, and other environmental measures are needed to explain trends and their sources. Moreover, comparing values from national biomonitoring programmes over time helps to indicate changes in sources of exposure, as well as the effectiveness of various regulatory measures.

Several time trend studies have attempted to evaluate the effectiveness of regulatory measures on levels of OH-PAHs. Hudson-Hanley et al. (2021) [27] investigated whether reductions in airborne PAH levels contributed to lower exposure to PAHs observed in the U.S. non-smoking population. Based on biomonitoring data from the National Health and Nutrition Examination Survey (NHANES) program covering the period 2001–2014, urinary concentrations of OH-NAPs and OH-PYR increased, while exposure to OH-FLUOs and OH-PHEs decreased, suggesting environmental sources of PAHs have changed over the time period (e.g., in ambient air) [27]. In China, a general decrease in urinary OH-PAH concentrations in children was noted between 2013 and 2016, which could be due to the implementation of pollution control. However, the sampling size was small [28]. Several New York City legislative regulations aimed at traffic-related air pollution may have contributed to reductions in the sum of 8 non-volatiles PAHs, particularly during the non-heating season, however urinary concentrations of 2-OH-NAP and 1-OH-PYR in children increased while 1-OH-NAP decreased [29]. Regulations related to smoking have partially reduced exposure to PAHs via passive inhalation for non-smokers in Germany after the 2000s [30]. Specifically, a decreasing trend for most measured OH-PAHs was observed from 1995 to 2019, except 2-hydroxy-naphthalene (2-OH-NAP), which increased during the same period [30].

Between our sampling periods 2011–2012 and 2019–2020, a complete ban on smoking on railway platforms, bus and tram stops, in restaurants, bars, zoos, and sports venues was approved in the Czech Republic (“Act No. 65/2017 Coll. Act on the Protection of Health from the Harmful Effects of Addictive Substances,” 2017) [31]. Also, the overall number of smokers in the population declined during this period, and there was a visible switch to e-cigarettes among smokers after the ban [32]. Another important measure is the 2012 Act on Air Protection (``Act No. 201/2012 Coll. Act on Air Protection,” 2012) [33], which can help reduce polycyclic aromatic hydrocarbons (PAHs) by regulating emissions and setting air quality standards to limit harmful pollutants (such as volatile organic compounds (VOCs), nitrogen oxides, sulphur dioxide, and particulate matter). The law also mandates regular monitoring, reporting, and the implementation of air quality improvement programs. It aligns with European Union regulations and aims to reduce the impact of chemical substances on human health and the environment. In addition, the Ministry of the Environment’s New Green Savings Programme (NGSP) was launched in 2014 to support the replacement of old boilers with greener alternatives, including emission-free technologies such as heat pumps and solar systems. Both legislative regulations and NGSP may have affected the overall exposure of the population and influenced the profile of OH-PAHs.

The aim of this study was to find out whether PAH metabolite levels in the Czech population were decreasing over time as a possible effect of regulatory measures such as the smoking ban (2017) and The Act on Air Protection (2012). Metabolic biomarkers of exposure to PAHs, i.e., hydroxylated (OH)-PAHs, were measured in urine samples collected in 2011–2012 from mothers and children within the DEMOCOPHES-CZ project and in 2019–2020 from children, adolescents and young adults within the CELSPAC (Central European Longitudinal Studies of Parents and Children) study and the periods were compared in terms of exposure to PAHs. Another goal was to establish reference values of PAH metabolites in urine for children, teenagers, and adults in the Czech Republic. The influence of determinants of OH-PAH exposure (including sex, education, smoking, consumption of different types of food, season, heating and cooking method, or degree of urbanisation) was also examined for age-specific categories.

## Methods

### Study population

Urinary metabolite biomarkers of exposure to PAHs, i.e., OH-PAHs, were analysed in samples of mothers and children from the same household from the DEMOCOPHES project and samples of children, teenagers and young adults from the CELSPAC (Central European Longitudinal Studies of Parents and Children) studies.

Czech DEMOCOPHES (DEMOCOPHES-CZ) was an integral part of the twin European human biomonitoring projects COPHES (Consortium to Perform Human Biomonitoring on a European Scale) and DEMOCOPHES [34], conducted from 2011 to 2012 by the National Institute of Public Health (NIPH Prague) of the Czech Republic [35]. In the Czech Republic, 120 pairs of children aged 6 to 11 years old and their mothers from urban and rural areas were examined, questionnaires were completed, and each participant donated one first-morning urine sample. The study was approved by the Ethical Committee of the NIPH Prague, and all mothers recruited gave informed written consent to participate in the study. The samples were collected at the same time for mothers and children from the same household. The study was approved by the ethics committee of the National Institute of Public Health (NIPH) no. 1464/2011, all mothers recruited gave informed written consent to participate in the study, and the data were handled according to the European and national legal and ethical requirements. From DEMOCOPHES-CZ a subset of 119 children and 116 mothers were used for analysis.

Two cohorts of the wider CELSPAC studies were included in this study:

The CELSPAC: Young Adults represents a follow-up study of the longitudinal ELSPAC study that was initiated as a birth cohort between 1991 and 1992 in the Czech Republic [36]. The CELSPAC: Young Adults follows ELSPAC children, their siblings, and spouses from 2019 to present, i.e., the study is ongoing. The study was approved by the CELSPAC ethics committee ELSPAC/EK/2/2019 and complies with European and national legal and ethical requirements. Each participant and parents or legal guardians provided written consent. A subset of 315 first morning urine samples were analysed from CELSPAC: Young Adults participants aged between 18 and 37 years between March and December 2019. Data for adults was provided within the HBM4EU (European Human Biomonitoring Initiative) aligned studies framework [37]. Questionnaires were administered to participants, including residential characteristics (degree of urbanisation, type of heating), diet (frequency of consumption of products), and smoking status (smokers, passive smokers, and non-smokers).

The CELSPAC: School Children and CELSPAC: Teenagers studies were conducted in schools of the South Moravian region in the Czech Republic between 2019 and 2020. The main aim of this cross-sectional studies was to assess the multiple factors potentially affecting the physical fitness of school children. All children recruited to the study completed questionnaires, underwent examinations, and donated the first-morning urine. The urine samples were collected between October 2019 and December 2019, except for one sample collected in January 2020. The studies were approved by the ethics committee EKV-2019-002 and EKV-2019-046 and complied with European and national legal and ethical requirements. Each parent or legal guardian of the participant provided written consent. The CELSPAC: School Children study included 195 children aged 10–11, and the CELSPAC Teenagers study included 299 children aged 12–17. Each CELSPAC participant donated one first-morning urine sample. General characteristics of the study populations are in Table 1.

**Table 1:** General characteristics of the study populations.

	DEMOCOPHES-CZ (2011–2012)				CELSPAC (2019-2020)		
Total number		Children	Mothers		School Children	Teenagers	Young Adults
		119	116		195	299	315
Age (years)	Median	8	37	Median	11	13	27
	5th-95th perc.	6–11	31–44	5^th^–95^th^ perc.	10 - 11	12 - 15	25 – 30
	Min. - Max.	6–11	28–47	Min. - Max.	10 - 11	12 - 17	18 - 37
Sex	female	60 (50.4%)	116 (100%)	Female	106 (54.4%)	126 (42.1%)	162 (51.4%)
	male	59 (49.6%)	0 (0%)	Male	89 (45.4%)	173 (57.9%)	153 (48.6%)
Sampling months	October-January	116 (97.5%)	113 (97.4%)	October-January	48 (24.6%)	299 (100%)	78 (14.1%)
	March-September	3 (2.5%)	3 (2.6%)	March-September	147 (75.4%)	0 (0%)	237 (85.9%)
Locality	Town/city/suburbs	58 (48.7%)	56 (48.3%)	Town/City	110 (56.4%)	126 (42.1%)	224 (71.7%)
	Village (rural)	60 (50.4%)	59 (50.9%)	Village (rural)	51 (26.2%)	149 (49.8%)	85 (27%)
	NA	0 (0%)	0 (0%)	NA	34 (17.4%)	24 (8.1%)	6 (1.9%)
Factory near	Yes	8 (6.7%)	8 (6.9%)				
	No	111 (93.3%)	108 (93.1%)				
Smoke in house (passive smoking)	Yes	13 (10.9%)	13 (11.2%)	Yes	0 (0%)	35 (11.7%)	61 (19.4%)
	No	106 (89.1%)	103 (88.8%)	No	0 (0%)	235 (78.6%)	238 (75.6%)
	NA	0 (0%)	0 (0%)	NA	195 (100%)	29 (9.7%)	16 (5.1%)
Smoking	Yes, daily	0 (0%)	13 (11.2%)	Yes	0 (0%)	1 (0.33%)	40 (12.7%)
	Yes, occasionally	0 (0%)	11 (9.5%)				
	No, I gave up smoking	0 (0%)	18 (15.5%)	No	195 (100%)	270 (90.03%)	261 (82.9%)
	No, I have never smoked	119 (100%)	74 (63.8%)				
	NA	0 (0%)	0 (0%)	NA	0 (0%)	28 (9.36%)	14 (4.4%)
Cooking on gas	Yes	58 (48.7%)	55 (47.4%)	Yes	84 (43.1%)	149 (49.8%)	158 (50.2%)
	No	61 (52.1%)	61 (52.6%)	No	81 (41.5%)	121 (40.5%)	142 (45.1%)
	NA	–	–	NA	30 (15.4%)	29 (9.7%)	15 (4.8%)
Cooking on coal or wood*	Yes	1 (0.8%)	1 (0.9%)				
	No	118 (99.2%)	115 (99.1%)				
Cooking with electricity*		Yes	81 (41.5%)	121 (40.5%)	142 (45.1%)		
		No	84 (43.1%)	149 (49.8%)	158 (50.2%)		
		NA	30 (15.4%)	29 (9.7%)	15 (4.8%)		
Type of heating	Coal/charcoal/wood	16 (13.4%)	16 (13.8%)	Coal/wood/ biomass	31 (15.9%)	75 (25.1%)	0 (0%)
	Other	66 (55.5%)	63 (54.3%)	Other	33 (16.9%)	65 (21.7%)	0 (0%)
	NA	37 (31.1%)	37 (31.9%)	NA	131 (67.2%)	159 (53.2%)	315 (100%)
Seafood and sea fish consumption	Daily	0 (0%)	0 (0%)	Daily	2 (1%)	1 (0.3%)	1 (0.3%)
	Several times a week	4 (3.4%)	10 (8.6%)	Several times a week (5-6x)	1 (0.5%)	2 (0.6%)	2 (0.6%)
				Several times a week (2-4x)	6 (3.1%)	10 (3.3%)	22 (7%)
	1 x a week	22 (18.5%)	28 (24.1%)	1 x a week	26 (13.3%)	68 (22.7%)	69 (21.9%)
	2-3 x a month	38 (31.9%)	32 (27.6%)	<3 x a month	89 (45.6%)	166 (55.5%)	193 (61.3%)
	1 x a month	25 (21%)	28 (24.1%)				
	Almost never	30 (25.2%)	18 (15.5%)	Never	36 (18.5%)	33 (11%)	18 (5.7%)
	NA	0 (0%)	0 (0%)	NA	35 (18%)	19 (6.4%)	10 (3.2%)
Fish consumption	Daily	1 (0.8%)	0 (0%)	Daily	2 (1%)	1 (0.3%)	1 (0.3%)
	Several times a week	7 (5.9%)	15 (12.9%)	Several times a week (5-6x)	2 (1%)	2 (0.7%)	2 (0.6%)
				Several times a week (2-4x)	8 (4.1%)	10 (3.3%)	22 (7.0%)
	1 x a week	25 (21%)	32 (27.6%)	1 x a week	33 (16.9%)	68 (22.7%)	69 (21.9%)
	2-3 x a month	45 (37.8%)	41 (35.3%)	<3 x a month	104 (53.3%)	166 (55.5%)	193 (61.3%)
	1 x a month	21 (17.6%)	18 (15.5%)				
	Almost never	20 (16.8%)	10 (8.6%)	Never	13 (6.7%)	32 (10.7%)	18 (5.7%)
	NA	0 (0%)	0 (0%)	NA	33 (16.9%)	20 (6.7%)	10 (3.2%)
Meat consumption	Daily	34 (28.6%)	30 (25.9%)	Daily	26 (13.3%)	53 (17.7%)	35 (11.1%)
	Several times a week	80 (67.2%)	69 (59.5%)	Several times a week (5-6x)	38 (19.5%)	57 (19.1%)	61 (19.4%)
				Several times a week (2–4x)	86 (44.1%)	153 (51.2%)	155 (49.2%)
	1 x a week	3 (2.5%)	13 (11.2%)	1 x a week	14 (7.2%)	11 (3.7%)	34 (10.8%)
	2-3 x a month	0 (0%)	1 (0.9%)	<3 x a month	0 (0%)	4 (1.3%)	7 (2.2%)
	Almost never	2 (1.7%)	3 (2.6%)	Never	0 (0%)	3 (1.0%)	13 (4.1%)
	NA	0 (0%)	0 (0%)	NA	31 (19.5%)	18 (6.0%)	10 (3.2%)
Offal consumption	Daily	0 (0%)	0 (0%)	Daily	0 (0%)	1 (0.3%)	1 (0.3%)
	Several times a week	0 (0%)	0 (0%)	Several times a week (5-6x)	0 (0%)	0 (0%)	0 (0%)
				Several times a week (2–4x)	1 (0.5%)	0 (0%)	2 (0.6%)
	1 x a week	2 (1.7%)	4 (3.4%)	1 x a week	3 (1.5%)	7 (2.3%)	9 (2.9%)
	2-3 x a month	7 (5.9%)	14 (12.1%)	<3 x a month	61 (31.3%)	90 (30.1%)	132 (41.9%)
	1 x a month	18 (15.1%)	32 (27.6%)				
	Almost never	92 (77.3%)	66 (56.9%)	Never	97 (49.7%)	179 (59.9%)	161 (51.5%)
	NA	0 (0%)	0 (0%)	NA	33 (16.9%)	22 (7.4%)	10 (3.2%)
Cereal consumption	Daily	60 (50.4%)	60 (51.7%)	Daily	105 (53.9%)	186 (62.2%)	127 (40.3%)
	Several times a week	34 (28.6%)	24 (20.7%)	Several times a week (5-6x)	48 (24.6%)	66 (22.1%)	65 (20.6%)
				Several times a week (2-4x)	11 (5.6%)	27 (9.0%)	96 (30.5%)
	1 x a week	5 (4.2%)	10 (8.6%)	1 x a week	0 (0%)	1 (0.3%)	10 (3.2%)
	2-3 x a month	5 (4.2%)	4 (3.4%)	<3 x a month	0 (0%)	0 (0%)	7 (2.2%)
	1 x a month	6 (5%)	9 (7.8%)				
	Almost never	8 (6.7%)	9 (7.8%)	Never	0 (0%)	0 (0%)	0 (0%)
	NA	0 (0%)	0 (0%)	NA	31 (15.9%)	19 (6.4%)	10 (3.2%)
Highest level of education**	Basic	3 (2.5%)	3 (2.6%)	Basic Secondary	0 (0%)	1 (0.3%)	3 (1%)
	Secondary	51 (42.9%)	49 (42.2%)		17 (8.7%)	134 (44.8%)	75 (23.8%)
	Higher	65 (54.6%)	64 (55.2%)	Higher	29 (14.9%)	136 (45.5%)	234 (74.3%)
	NA	0 (0%)	0 (0%)	NA	149 (76.4%)	28 (9.4%)	3 (1%)

*NA* values not filled in by participants; *not available for CELSPAC or DEMOCOPHES-CZ cohort; **for DEMOCOPHES-CZ: the highest education in household; Locality (CELSPAC): Village (rural) < population = 3000 > = Town/City; Factory near: factory within a 50 m radius around home; Seafood consumption: fresh and frozen seafood; Fish consumption: fresh, frozen and preserved sea and freshwater fish; Meat consumption: all kinds of meat and meat products (except offal); Cereal consumption: bread, sweet and savoury pastry, oatmeal and other cereal products; Highest level of education: Basic (ISCED < 3), Secondary (3 < = ISCED < 5), Higher (ISCED > = 5), classified based on the International Standard Classification of Education (ISCED) [77]. Frequency of consumption of a particular food category was determined as the frequency of the most frequently consumed item.

### Chemical analysis

The concentrations of OH-PAHs were determined using liquid chromatography with tandem mass spectrometry (LC-MS/MS). Prior to instrumental analysis, enzymatic hydrolysis and solid phase extraction (SPE) were used for metabolite deconjugation and clean-up. The general methodology is based on CDC Laboratory Procedure Manual 6703.04 [38].

Each batch of samples contained a blank matrix, SRM QC material (NIST SRM 3672 and 3673), and in-house QC samples. Quantification of OH-PAH was done via the stable isotope dilution method. Matrix blank was subtracted for each analyte per batch to eliminate the background and noise contribution to the analyte concentration. Quality control is ensured by successful participation in the HBM4EU quality assurance (QA)/QC programme [39] with four rounds of Inter-laboratory Comparison Investigations (ICIs) and External Quality Assurance Schemes (EQUASs) [40]. Full methodological details are reported in Supplementary Material and Tables S1 and S2.

### Statistical analysis

Values below LOD were substituted with LOD/√2. Analyte concentrations were adjusted to creatinine and specific gravity, except DEMOCOPHES-CZ samples where specific gravity was not available.

Spearman’s correlation was used to determine the relationship among OH-PAHs. In the case of values < LOD, pairwise deletion was performed before the analysis. 3-OH-BaP showed a detection frequency below 5% for CELSPAC and 20% for DEMOCOPHES and was excluded from the analysis.

For statistical analyses, individual metabolites and the ΣOH-NAP (sum of 1-OH-NAP, 2-OH-NAP), ΣOH-FLUO (sum of 2-OH-FLUO and 3-OH-FLUO) and ΣOH-PHE (sum of 1-OH-PHE, 2/3-OH-PHE, 4-OH-PHE, 9-OH-PHE) were used.

Summary statistics of each metabolite concentration were calculated (min, max, median, geometric mean, and percentiles). The reference value for a chemical substance in biological material (e.g., blood, urine) is derived using a defined statistical method [41] from measurements taken from a specific population group. Reference values derived as the 95^th^ percentile of the measured pollutant concentration in the relevant matrix of the reference population allow comparing the exposure of individuals or population groups to the exposure of the general population, at local or regional scales [42]. As environmental conditions change, reference values are regularly reviewed and updated. Reference values RV_95_ of CELSPAC cohorts (2019-2020) were calculated, and their 95% confidence intervals were derived using the bias-corrected and accelerated bootstrap (BCa) procedure [43]. Urine samples with creatinine concentrations <0.3 or >3.0 g/L were excluded before the calculation of RV_95_ [44].

Nonparametric Mann-Whitney U test and Kruskal-Wallis test with Dunn´s post-hoc test were used to test differences in individual and summed PAH metabolite concentrations between sex, time period, and age categories as the first step.

Multiple linear regression models were applied to evaluate the association between the OH-PAH metabolite levels and the exposure factors from questionnaires (Table 1). Concentrations were transformed by natural logarithm, and the outliers were excluded before analysis (by applying the interquartile (IQR) method). The normal distribution of residuals was checked by using histograms and the Kolmogorov-Smirnov test. Regression models for the 1-OH-NAP, 2-OH-NAP, ΣOH-NAP, ΣOH-FLUO, and ΣOH-PHE and 1-OH-PYR were computed with all significant exposure factors to determine the maximum explained variability (Coefficient of determination, R^2^). Regression analyses were performed for each cohort and age group DEMOCOPHES-CZ (children, mothers) and CELSPAC (children, teenagers) separately.

Data analyses and visualisation were performed using R project (version 4.3.2) and Statistica (version 13.5.0.17).

## Results

Nine of the 10 measured OH-PAHs were detected in the DEMOCOPHES-CZ and CELSPAC samples (Table S2). 1-OH-NAP, 2-OH-NAP, 2-OH-FLUO, and 2/3-OH-PHE were detected with the highest frequency above 90% in all cohorts. 3-OH-FLUO and 1-OH-PYR were detected with a frequency between 75% and 97.5%, 1-OH-PHE and 4-OH-PHE between 66.7% and 99.5%. 9-OH-PHE was detected with a low frequency of 9.2% for children and 45.2% for teenagers from cohort CELSPAC, and 3-OH-BaP was detected in DEMOCOPHES-CZ cohort with a frequency of 11.2% for mothers and 7.6% for children. 3-OH-BaP was only detected in 1% of samples of teenagers and not detected in children, nor young adults from CELSPAC.

**Fig. 1:**
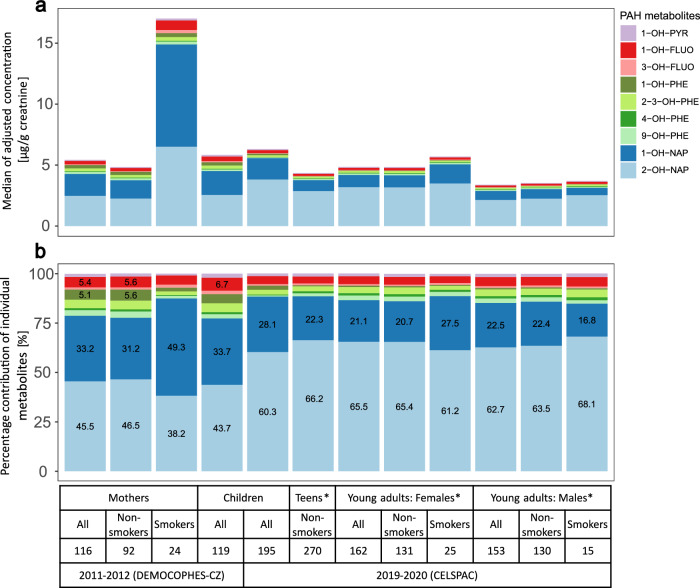
Contribution of metabolites to the total ΣOH-PAH. The median **a** and percentage **b** contribution of individual metabolites to the total ΣOH-PAH in each age group and sampling period. *14 participants from CELSPAC young adults and 28 participants from CELSPAC teenagers did not answer questions about smoking; only one participant from CELSPAC teenagers was a smoker.

**Fig. 2:**
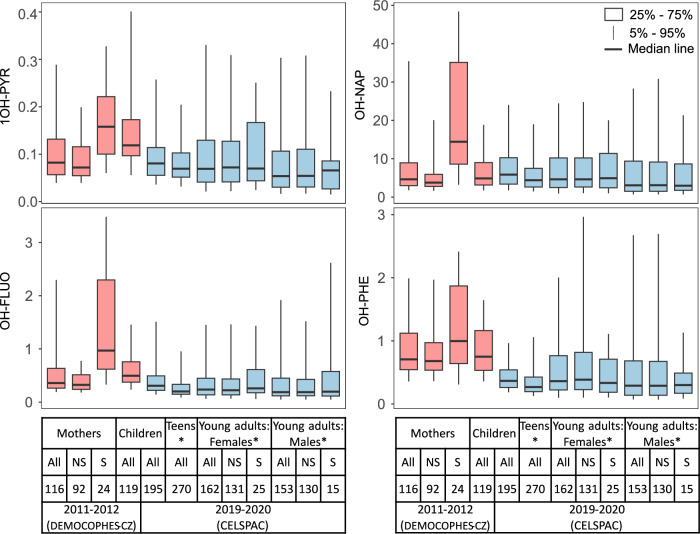
Comparison of OH-PAH metabolite concentrations (µg/g creatinine) between cohorts (DEMOCOPHES-CZ and CELSPAC) and sampling years. Sums are given for ΣOH-NAPs, ΣOH-PHEs, and ΣOH-FLUOs. *NS* means non-smokers, *S* means smokers, and *All* indicates the total number of participants in the cohort. *14 participants from CELSPAC young adults and 28 participants from CELSPAC teenagers did not answer questions about smoking; only one participant from CELSPAC teenagers was a smoker.

OH-PAH metabolites are correlated with each other in all cohorts (Fig. S1a–e). As expected, the highest correlations are among metabolites that originate from the same parent compound. 1-OH-NAP and 2-OH-NAP showed high correlations with ΣOH-PAH, r_s_ = 0.99 and r_s_ = 0.81 respectively. Due to their high concentration contribution, ΣOH-PAH mainly reflects the pattern of ΣOH-NAP (Figs. 1, 2).

### Levels of PAH metabolites in urine

The analytes with the highest median concentrations in all cohorts were 2-OH-NAP (2.5–3.8 µg/g creatinine), followed by 1-OH-NAP (0.9–1.9 µg/g creatinine). The contribution of 1-OH-NAP and 2-OH-NAP to the total ΣOH-PAH ranges from 80.3–93.5%, whilst the concentrations of the other OH-PAHs were an order of magnitude lower. The median concentrations of ΣOH-PHE were between 0.27 and 0.75 µg/g creatinine, and ΣOH-FLUO were between 0.2 and 0.5 µg/g creatinine, with contribution to ΣOH-PAH being 5.7–13% and 4.3–8.5%, respectively. The median concentration of 1-OH-PYR, commonly used as a marker of the high molecular weight PAHs, was in the range of 0.062–0.119 µg/g creatinine, representing only 1.3–2% of the total sum of metabolites (Fig. 1). The levels of urinary PAH metabolites (1-OH-NAP, 2-OH-NAP, 2-OH-FLUO, 3-OH-FLUO, 1-OH-PHE, 2/3-OH-PHE, 4-OH-PHE, 9-OH-PHE, 1-OH-PYR, and 3-OH-BaP) and their sums according to the parent compound, for each age group and sampling period, are presented in Table 2 (creatinine-adjusted concentrations) and Tables S3–S4 (non-adjusted /SG standardised concentrations).

**Table 2:** Descriptive statistics of urinary PAH metabolite concentrations (µg/g creatinine) measured in DEMOCOPHES and CELSPAC cohorts.

Cohort study / Population	Analyte	Creatinine adjusted (μg/g creatinine)							
		Min	5 P	25 P	Median	GM	75 P	95 P	Max
DEMOCOPHES-CZ CHILDREN	1-OH-NAP	<LOD	0.736	1.271	1.968	2.411	4.001	9.902	137.3
	2-OH-NAP	0.577	1.042	1.615	2.549	2.716	4.198	8.484	15.813
	2-OH-FLUO	0.119	0.200	0.303	0.392	0.425	0.605	1.171	3.104
	3-OH-FLUO	<LOD	0.031	0.064	0.097	0.099	0.156	0.314	0.659
	1-OH-PHE	0.066	0.117	0.183	0.266	0.274	0.399	0.659	1.914
	2/3-OH-PHE	0.090	0.121	0.184	0.263	0.275	0.403	0.707	1.802
	4-OH-PHE	<LOD	0.022	0.038	0.061	0.061	0.087	0.188	0.481
	9-OH-PHE	<LOD	<LOD	0.079	0.118	0.124	0.177	0.398	0.829
	1-OH-PYR	<LOD	<LOD	0.096	0.119	0.130	0.173	0.403	0.580
	3-OH-BaP	<LOD	<LOD	<LOD	<LOD	<LOD	<LOD	0.161	0.250
	ΣOH-NAP	1.010	1.728	3.153	4.872	5.552	9.003	20.648	138.876
	ΣOH-FLUO	0.131	0.231	0.375	0.496	0.530	0.766	1.464	3.420
	ΣOH-PHE	0.249	0.348	0.532	0.749	0.767	1.165	1.811	4.094
DEMOCOPHES-CZ Mothers	1-OH-NAP	<LOD	0.700	1.171	1.804	2.371	3.964	21.293	67.979
	2-OH-NAP	<LOD	0.847	1.617	2.474	2.895	4.434	15.942	30.054
	2-OH-FLUO	0.108	0.155	0.220	0.291	0.359	0.504	1.687	3.089
	3-OH-FLUO	<LOD	<LOD	0.039	0.061	0.071	0.119	0.533	1.085
	1-OH-PHE	<LOD	0.108	0.195	0.278	0.282	0.395	0.739	1.686
	2/3-OH-PHE	<LOD	0.097	0.157	0.232	0.241	0.338	0.692	0.912
	4-OH-PHE	<LOD	<LOD	0.038	0.056	0.060	0.095	0.199	0.386
	9-OH-PHE	<LOD	<LOD	0.099	0.155	0.155	0.220	0.728	3.281
	1-OH-PYR	<LOD	<LOD	0.057	0.082	0.087	0.132	0.292	0.400
	3-OH-BaP	<LOD	<LOD	<LOD	<LOD	<LOD	<LOD	0.169	0.229
	ΣOH-NAP	1.192	1.750	3.001	4.627	5.582	8.929	36.56	72.389
	ΣOH-FLUO	0.121	0.185	0.262	0.358	0.436	0.637	2.302	4.174
	ΣOH-PHE	0.207	0.354	0.542	0.706	0.791	1.130	2.008	4.482
CELSPAC: YOUNG ADULTS	1-OH-NAP	<LOD	<LOD	0.404	0.916	1.007	2.154	10.981	232.552
	2-OH-NAP	<LOD	0.510	1.225	2.864	2.860	6.715	17.462	159.532
	2-OH-FLUO	<LOD	0.044	0.103	0.178	0.205	0.368	1.186	17.223
	3-OH-FLUO	<LOD	<LOD	0.017	0.036	0.041	0.083	0.416	7.381
	1-OH-PHE	<LOD	<LOD	<LOD	0.029	0.034	0.055	0.217	3.943
	2/3-OH-PHE	<LOD	<LOD	0.075	0.132	0.142	0.254	0.893	12.977
	4-OH-PHE	<LOD	<LOD	<LOD	0.050	0.048	0.116	0.498	8.016
	9-OH-PHE	<LOD	<LOD	<LOD	0.092	0.094	0.266	0.838	12.491
	1-OH-PYR	<LOD	<LOD	0.034	0.062	0.068	0.120	0.327	6.463
	3-OH-BaP	<LOD	<LOD	<LOD	<LOD	<LOD	<LOD	<LOD	<LOD
	ΣOH-NAP	0.226	0.765	1.847	3.946	4.125	9.889	26.503	392.085
	ΣOH-FLUO	0.017	0.053	0.124	0.217	0.251	0.452	1.534	24.605
	ΣOH-PHE	0.035	0.083	0.176	0.324	0.364	0.727	2.593	30.041
CELSPAC: TEENAGERS	1-OH-NAP	<LOD	0.297	0.615	1.050	1.184	1.907	6.579	36.911
	2-OH-NAP	<LOD	1.013	1.880	3.131	3.302	5.556	13.098	62.983
	2-OH-FLUO	<LOD	0.048	0.113	0.165	0.182	0.272	0.805	4.154
	3-OH-FLUO	<LOD	<LOD	0.028	0.043	0.050	0.077	0.244	1.034
	1-OH-PHE	<LOD	<LOD	<LOD	0.027	0.030	0.043	0.105	0.740
	2/3-OH-PHE	<LOD	<LOD	0.080	0.115	0.124	0.168	0.4	2.341
	4-OH-PHE	<LOD	<LOD	0.029	0.047	0.044	0.067	0.12	1.034
	9-OH-PHE	<LOD	<LOD	<LOD	<LOD	0.082	0.182	0.458	5.048
	1-OH-PYR	<LOD	<LOD	0.052	0.069	0.075	0.103	0.208	1.382
	3-OH-BaP	<LOD	<LOD	<LOD	<LOD	<LOD	<LOD	<LOD	1.029
	ΣOH-NAP	0.401	1.469	2.641	4.507	4.715	7.595	19.173	76.906
	ΣOH-FLUO	0.025	0.087	0.147	0.202	0.238	0.335	0.979	5.016
	ΣOH-PHE	0.076	0.126	0.194	0.265	0.305	0.425	1.049	7.885
CELSPAC: SCHOOL CHILDREN	1-OH-NAP	<LOD	0.575	1.043	1.775	1.848	3.051	8.832	19.662
	2-OH-NAP	0.781	1.141	2.322	3.807	4.046	6.821	15.224	25.392
	2-OH-FLUO	0.071	0.120	0.183	0.258	0.288	0.407	1.039	2.079
	3-OH-FLUO	<LOD	<LOD	0.034	0.050	0.060	0.089	0.310	0.782
	1-OH-PHE	<LOD	0.051	0.092	0.130	0.138	0.195	0.388	0.856
	2/3-OH-PHE	<LOD	<LOD	0.103	0.152	0.162	0.246	0.482	1.190
	4-OH-PHE	<LOD	<LOD	<LOD	0.026	0.029	0.044	0.120	0.697
	9-OH-PHE	<LOD	<LOD	<LOD	<LOD	<LOD	<LOD	0.105	0.394
	1-OH-PYR	<LOD	<LOD	0.055	0.081	0.083	0.114	0.258	0.703
	3-OH-BaP	<LOD	<LOD	<LOD	<LOD	<LOD	<LOD	<LOD	<LOD
	ΣOH-NAP	1.137	1.763	3.374	5.863	6.029	10.264	24.612	37.556
	ΣOH-FLUO	0.085	0.140	0.221	0.308	0.353	0.497	1.556	2.430
	ΣOH-PHE	0.129	0.184	0.261	0.366	0.389	0.544	0.996	2.347

Values below LOD were replaced by LOD/√2. *LOD* limit of detection, *GM* geometric mean.

### Age and time-related trends in urinary OH-PAH levels

Children had significantly higher concentrations (µg/g creatinine) (KW, *p* < 0.05) of 1-OH-NAP, 2-OH-FLUO, 3-OH-FLUO, 2/3-OH-PHE, and 1-OH-PYR than nonsmoking mothers/adults and/or teenagers sampled in the same year (Fig. S2). The teenagers and young adults sampled in the same year (CELSPAC) had comparable concentrations of all measured OH-PAH metabolites except for 3-OH-FLUO and 2/3-OH-PHE (Fig. S2).

**Fig. 3:**
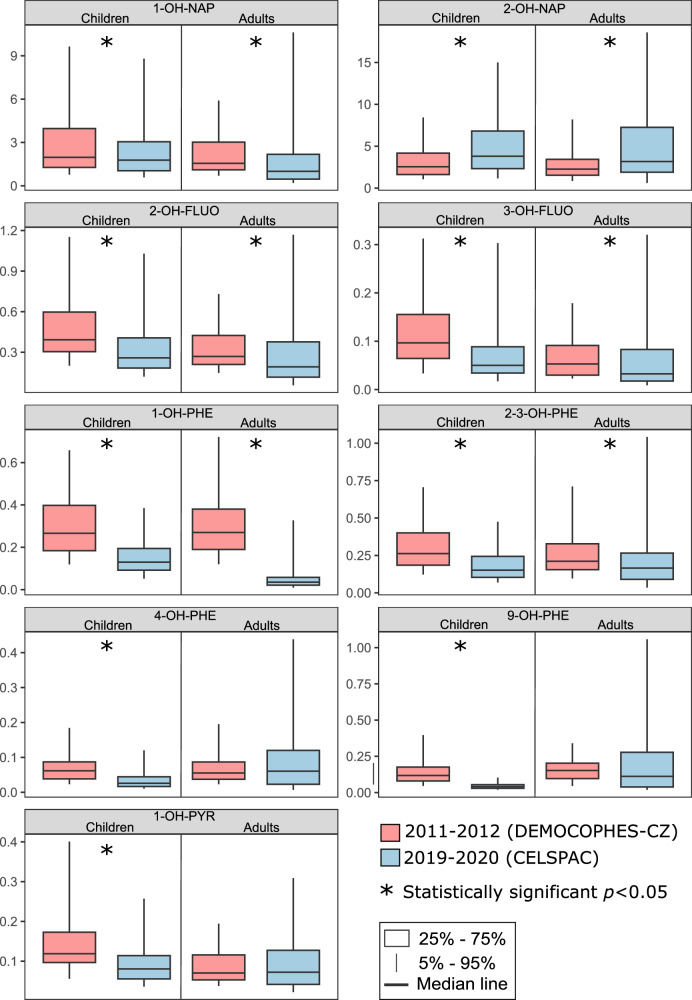
Comparison of OH-PAH metabolite concentrations (µg/g creatinine) between DEMOCOPHES-CZ and CELSPAC women (adults) and children in two sampling periods. Smokers were excluded.

Nearly ten years passed between the sampling periods, the changes in PAH metabolite concentrations between the two periods were tested for statistical significance. When removing the effects of sex (only females), smoking (only non-smokers), education (only middle and high), and age (25–44 years), a statistically significant difference in concentrations (MW, p < 0.05) with a median decrease of 35% for 1-OH-NAP, 35% for 2-OH-FLUO, 30% for 3-OH-FLUO, 85% for 1-OH-PHE, and 44% for 2/3-OH-PHE, and an increase of 29% in the median concentration of 2-OH-NAP was found in the second period (Figs. 3 and S3). The same results were obtained when the effect of season was also removed (not shown). Significantly declining creatinine-adjusted concentrations (MW, *p* < 0.05) between 10% and 51% of all measured metabolites and increasing concentrations of 2-OH-NAP by about 49% were also found in children from the second period compared to children from the first period (Figs. 3 and S3).

The largest differences in PAH metabolite concentrations between sampling periods were found in smokers (adjusted for gender) with a significant decrease (MW, *p* < 0.05) of all metabolites. The median concentration of 1-OH-NAP, OH-FLUOs, and 1-OH-PHE decreased several times with the median percentage decrease ranging from 76% to 91% (Figs. 3 and S3). The median percentage decrease ranged from 25% to 59% for 2-OH-PHEs, 3-OH-PHEs, 4- and 9-OH-PHEs, 2-OH-NAPH, and 1-OH-PYR. No statistically significant difference was found between smokers and non-smokers in the second sampling period (Fig. 1). The differences in PAH metabolite concentrations for age and between periods are similar for- non-adjusted concentrations (Figs. S4–S5).

Comparing the sampling periods, it can be summarised that a statistically significant decrease was found for all metabolites and the sum of metabolites of individual parent PAHs including OH-NAPs in smokers. There was also a statistically significant decrease in all metabolites (except 2-OH-PAH) in children. In non-smokers, the levels of 1-OH-NAPs, 2-OH-FLUO, 3-OH-FLUO, 1-OH-PHE and 2/3-OH-PHE decreased, while 2-OH-NAPs increased. ΣOH-PHE and ΣOH-FLUO decreased significantly in both children and adult nonsmokers (Fig. S6). These results suggest a reduction in exposure to phenanthrene and fluoranthene in both nonsmokers and children, and to pyrene in children. Regarding ΣOH-NAP, there was no significant change in the sum of the concentrations of the two metabolites between the periods in children and non-smokers, only a change in the ratio (Figs. 1 and  2), which is discussed in Section “Variation in 1-OH-NAP and 2-OH-NAP ratio”.

### Determinants of PAH exposure

Based on the results of time and age comparisons, cohorts and age groups were analysed separately. The analysis was performed for the sums of parent metabolites (creatinine-adjusted), i.e., ΣOH-NAPs, ΣOH-PHEs, and ΣOH-FLUOs, due to the high correlation among individual metabolites belonging to a given parent compound (Figs. S1a–e), and 1-OH-PYR. The CELSPAC: Young Adults dataset was part of the HBM4EU project and, thus, has not been assessed here.

Regression analysis showed smoking, fish and offal consumption, season, locality, and type of cooking were significant factors for any of the metabolites (Table S5). Other factors included in the analysis, such as sex, education, proximity of the factory to the house, type of heating, consumption of cereals and meat, and passive smoking, were not significant.

In the first sampling period (DEMOCOPHES-CZ study), the most significant factor was the mother’s smoking, with an increasing number of cigarettes per day associated with increasing concentrations of all PAH metabolites (Table S5). Mothers who smoked daily had two times higher median concentration of urinary OH-PAHs compared to mothers who smoked occasionally, former smokers, or mothers who did not smoke at all. Apart from smoking, no other significant factors were found for urinary concentrations of ΣOH-FLUOs, 1-OH-PYR, and ΣOH-PHEs in mothers.

Fish consumption of mothers (frequency as a number of days per week; including freshwater, sea fish, and other sea products) contributed about 5% to the explained variability of ΣOH-NAPs in DEMOCOPHES-CZ mothers and children (model for non-smokers), with higher fish consumption increasing ΣOH-NAP concentrations. Higher concentrations of ΣOH-PHEs were associated with a higher frequency of offal consumption in teenagers (CELSPAC). However, fish, seafood, and offal are consumed very little in the Czech population (Table 1).

Another significant factor for the ΣOH-NAPs concentration in mothers and children from DEMOCPHES-CZ and for all metabolites in CELSPAC children was the location (city vs. rural area) (Table S5). The concentrations were lower in people living in cities in 2011 and in people living in urban areas in 2019.

Significantly higher concentrations were found for ΣOH-NAPs and ΣOH-PHEs in the cold season compared to the warm season (May-Sep and cold period Oct-March). Lower concentrations of 1-OH-PYR and ΣOH-PHEs in CELSPAC children and teenagers were associated with the use of an electric appliance during cooking compared to gas cooking.

The regression models for mothers with smoking included explained a large amount of variability (43–45% for ΣOH-NAPs and ΣOH-FLUOs, with 26% for 1-OH-PYR), while the models for non-smokers (children and teenagers) with one or more statistically significant exposure factors like fish and offal consumption, season, locality, and type of cooking explained for a small amount of variability between 4 and 8% (Table S5).

A statistically significant correlation (*r* = 0.34–0.46) was found for OH-PAH metabolites between DEMOCOPHES-CZ children and mothers, indicating partial co-exposure due to living in the same household (Table S6). Given the inconclusiveness of the effect of smoking by household members on OH-PAH concentrations in children, it can be assumed that this is more likely an indoor air and dietary exposure. Although children spend time at school during the week (where they also eat) while parents are at work, the effect of exposure from one household is significant. Pairwise testing of the difference in concentrations between mothers and children confirmed the results of elevated concentrations of ΣOH-FLUOs, 1-OH-PYR, and OH-NAP metabolites in children from the age comparison (Table S6).

### Reference values

The reference value for a chemical substance in biological material (e.g., blood, urine) is calculated using a statistical method based on measurements from a specific population group, enabling comparison of individual or group exposure to background levels [42]. Reference values were calculated for children, teenagers, and young adults from the 2019-2020 CELSPAC cohort (Table 3). Smokers and urine samples with creatinine <0.3 or >3.0 g/L were excluded from the analysis. As no statistically significant sex difference was found for individual metabolites, reference values are given for each age category for both males and females combined. All the recommendations, including the exclusion (smoking) and partitioning (age) criteria, a minimum sample size of 120 people, the most recent biomonitoring data, and the sufficiency and quality of the analytical methods for the calculation of reference values, were followed [45]. A limiting factor is the lack of representative sampling from the whole country because the CELSPAC cohort only sampled the South Moravian region. However, the study participants do not differ in ethnicity, dietary habits, or other lifestyle factors from the general population, and they are not occupationally exposed. Consequently, it is supposed that the region is comparable to the rest of the country in terms of PAH exposures, and the proposed values can serve as indicative reference values. The calculated RV_95_ for children is limited by the small age range (10–11 years) of participants. Reference values are strictly statistically derived values that have no explicit health significance, by contrast to the health-based human biomonitoring guidance values (HBM GV) derived from toxicological and epidemiological studies to estimate the risk in a given population. PAHs are considered to be no-threshold carcinogens, and their concentrations should be as low as possible. Consequently, there are no health-based HBM guidance values for a quantitative assessment of risk to the population.

**Table 3:** Reference values RV_95_ and corresponding confidence interval CI of PAH metabolite concentrations for non-smoking children, teenagers and adults from CELSPAC cohort (2019–2020).

Unit	Age (years)		*N*	1-OH-NAP	2-OH-NAP	2-OH-FLUO	3-OH-FLUO	1-OH-PHE	2/3-OH-PHE	4-OH-PHE	9-OH-PHE	1-OH-PYR
Non-adjusted µg/L	CH	10-11	194	12 (7.37-19.8)	20 (15.4-25.9)	1.4 (1-2.31)	0.43 (0.3-0.61)	0.53 (0.43-0.74)	0.69 (0.6-0.91)	0.17 (0.1-0.21)	0.10 (0.06-0.15)	0.36 (0.3-0.39)
	TE	12-17	254	10 (7.27-16.2)	22 (18.3-29)	1.1 (0.78-1.56)	0.37 (0.26-0.56)	0.15 (0.09-0.16)	0.61 (0.44-0.74)	0.16 (0.13-0.17)	0.74 (0.52-1.16)	0.30 (0.24-0.44)
	A	18-37	237	9.6 (7.31-14.6)	20 (16.7-26.8)	1.5 (0.98-1.96)	0.41 (0.26-0.67)	0.29 (0.17-0.53)	0.85 (0.59-1.33)	0.64 (0.29-0.93)	0.91 (0.62-1.23)	0.28 (0.24-0.31)
Creatinine-adjusted µg/g	CH	10-11	194	8.8 (6.38-15.1)	15 (12.5-20.8)	1.0 (0.86-1.47)	0.31 (0.26-0.4)	0.39 (0.32-0.63)	0.49 (0.40-0.74)	0.12(0.09-0.19)	0.11 (0.09-0.13)	0.26 (0.19-0.41)
	TE	12-17	254	6.8 (4.76-11)	13 (10.4-18.2)	0.77 (0.57-0.96)	0.26 (0.18-0.33)	0.088 (0.06-0.12)	0.36 (0.28-0.43)	0.12 (0.1-0.13)	0.46 (0.34-0.59)	0.20 (0.15-0.23)
	A	18-37	237	11 (7.08-14.7)	18 (13.6-20.7)	1.2 (0.92-2.32)	0.40 (0.26-0.51)	0.23 (0.15-0.38)	0.95 (0.54-1.34)	0.49 (0.27-0.78)	1.0 (0.63-1.41)	0.32 (0.24-0.35)

CH - children, TE - teenagers, A - adults. Non-adjusted (µg/L) and creatinine-adjusted (µg/g creatinine) concentrations were used.

Summary statistics of creatinine-adjusted and non-adjusted PAH metabolite concentrations are in the supplementary material, including the 95^th^ percentile for smoking and non-smoking mothers from the DEMOCOPHES-CZ (Tables S7 and S8, respectively) and for all age groups from CELSPAC cohort (Table S9).

## Discussion

### Time pattern and legislative changes

#### OH-PAH levels and smoking regulation

Compared to 2011–2012, non-smoking adults and children in 2019–2020 showed lower levels of 1-OH-NAP, 2-OH-FLUO, 3-OH-FLUO, 1-OH-PHE, and 2/3-OH-PHE; among children, concentrations of 4-OH-PHE, 9-OH-PHE, and 1-OH-PYR also decreased, while levels of 2-OH-NAP increased in both children and adults (Fig. 1, Fig. S3). The similar pattern of a significant decrease in urinary 3-OH-BaP, 1-OH-PYR, 1-OH-NAP, 1-, 2-, and 3-OH-PHE, along with an increase in 2-OH-NAP, over time, was observed in a non-smoking population in Germany, in a study focused on smoking regulations [30]. Starting in 2002, Germany introduced regulations to reduce secondhand tobacco smoke (SHS) exposure. The impact of regulations on PAH and SHS exposure was assessed by analyzing cotinine, a metabolite of nicotine, and selected OH-PAHs in 510 urine samples collected from 1995 to 2019. The results showed a significant correlation of cotinine with 1-OH-PYR, 1-OH-NAP, and all OH-PHE metabolites (but no correlation was found for 2-OH-NAP), 82% decline in urinary cotinine levels and significant decrease in several OH-PAHs in urine, including 1-OH-PYR, 1-OH-NAP, 1-, 2- and 3-OH-PHE over the 24-year period. Declines were attributed to smoking bans and regulations limiting SHS and PAH exposure [30]. These findings align with earlier results from the US National Health and Nutrition Examination Survey (NHANES) conducted in children and adolescents, which revealed a correlation between SHS, measured as serum cotinine levels, and OH-NAP exposure between 2003 and 2008 [46]. Additionally, smokers, both adults and teenagers, as well as individuals exposed to SHS from NHANES in 1999–2000, had urinary 1-OH-PYR levels 2-3 times higher than non-smokers in the same age group (other metabolites were not tested) [13]. Between our sampling periods 2011–2012 and 2019–2020, a complete ban on smoking on railway platforms, bus and tram stops, in restaurants, bars, zoos, and sports venues was approved in the Czech Republic [31]. According to the National Institute of Public Health (NIPH), the total number of smokers in the Czech population decreased during the sampling period, with a significant shift towards electronic cigarettes after the smoking ban [32]. A lower proportion of smokers in 2019 (12.7%) compared to 2011 (20.7%) is also visible in our study. While smokers in 2011–2012 had three times higher median concentration of OH-PAHs compared to non-smokers, by 2019, there was no longer a difference between smokers and non-smokers (Fig. 1). According to the questionnaires, the average number of cigarettes smoked per day decreased from 5.9 in 2011–2012 to 3.8 in 2019. In 2019, there were more occasional smokers (52.5% of all smokers compared to 16.6% in 2011–2012), and 20% of participants reported using both electronic and regular cigarettes. Exposure to PAHs from e-cigarettes has been found to be significantly lower than from combustible cigarettes, with concentrations of OH-PAHs in e-cigarette users (or users of other tobacco/nicotine products, including heated tobacco, oral tobacco, and nicotine replacement therapy) comparable to nonsmokers [47]. The decline in all OH-PAH metabolites among smokers and most metabolites in nonsmokers over the 10-year period suggests that smoking bans, reduced smoking rates, and switching to e-cigarettes likely contributed to the reduction in PAH exposure in the population. The increase in 2-OH-NAP is further discussed in Section “Variation in 1-OH-NAP and 2-OH-NAP ratio”.

#### OH-PAH levels and air pollution

Another route of exposure to PAHs is inhalation from the outdoor air. Daily intake from inhalation is between 1 and 28% (most commonly around 10%, depending on air pollution in the area), compared to dietary exposure, which contributes up to 90% of total daily intake [48–51].

Factors that may be associated with exposure to PAHs from ambient air are place of residence (location) and seasonality. In the present study, higher concentrations were found in the cold season (October–March). Seasonality was only tested for CELSPAC children as the DEMOCOPHES-CZ study and CELSPAC teenagers were sampled predominantly in the autumn and winter months (Table 1). The elevated concentrations of PAHs during the cold season may be primarily related to local heating. Seasonal trends, higher concentrations of NAP, FLUO, PHE, PYR, and BaP in the air in the cold season, are also visible at measuring stations in the Czech Republic and Europe in both urban and background areas [52, 53]. Locality was a significant factor in the 2011 sampling, with children in urban areas showing lower 1-OH-NAP and 2-OH-NAP levels (cold season). In contrast, children from 2019 had lower concentrations in villages, possibly due to the impact of the New Green Savings Programme from 2014. According to the Czech Hydrometeorological Institute (CHMI), the reduction in emissions in recent years has also been influenced by the replacement of older boilers, the switch to natural gas, and the use of non-emission sources, particularly heat pumps [54]. Seasonality and locality explained 4–7% of the total variability of OH-PAHs in CELSPAC children (Table S5).

The Act on Air Protection in the Czech Republic came into effect in 2012 [33]. A noticeable reduction in air PAH concentrations occurred between 2010 and 2020. Annual median values showed significant decreases in fluorene, phenanthrene, and naphthalene concentrations from 2011 to 2020 at the Košetice EMEP station, with similar trends observed in the cities/towns where the participants live [55]. These findings are consistent with the observed differences in metabolite concentrations between the DEMOCOPHES-CZ and CELSPAC children and suggest a possible relationship between the concentration of PAHs in the air and the concentration measured in urine. However, a more detailed analysis with the inclusion of geocoded data, including, for example, distance from industrial areas or road density, would be needed to explain these relationships. Details about trends in PAHs in the air are provided in the Supplementary Material and Table S10.

A more pronounced decrease in PAH metabolites (i.e. 1-OH-NAP, 2/3-OH-FLUO, and 1-OH-PYR) was observed in smokers, whereas the reduction among non-smokers was considerably smaller (Fig. S3). These trends likely reflect a combination of factors, including air quality legislation, the public smoking ban, and possible changes in smoking behaviour.

### OH-PAH levels and diet and food preparation

Another determinant of exposure in the non-smoking population without occupational exposure is diet and food preparation [56]. The main pathway is the formation of PAHs during industrial food processing (e.g., drying, roasting) or home food preparation (e.g., grilling, roasting, frying, smoking) at high temperatures [57]. Higher levels of PAHs are mainly present in high-fat products and smoked or grilled meat and fish products [58–60]. Also, marine species, mainly bivalves, contain high PAH concentrations [61]. Dietary exposure particularly increases levels of low molecular weight, less toxic PAHs compared to high molecular weight PAHs [62, 63]. In the present study, there were significant relationships between fish consumption and ΣOH-NAPs concentrations in DEMOCOPHES-CZ mothers and children and between offal consumption and ΣOH-PHEs concentrations in CELSPAC teenagers (Table S5). The questionnaires lack some details that may be interesting for the Czech population, such as the consumption of pork; and food processing, such as smoking. However, the CELSPAC questionnaires provided data on the use of gas or electricity for cooking. Concentrations of ΣOH-PHEs and 1-OH-PYR in CELSPAC children and teenagers were negatively associated with the use of electricity for cooking and baking and positively associated with the use of gas or gas/electricity for cooking (Table S5). The use of natural gas appliances and heating was identified as one of the major sources of PAHs in non-smoking households [64]. However, regression models for non-smokers, including fish and offal consumption and/or cooking type, explain only a small amount of the 4–7% variability in urinary PAH metabolite concentrations (Table S5).

### Variation in 1-OH-NAP and 2-OH-NAP ratio

**Fig. 4:**
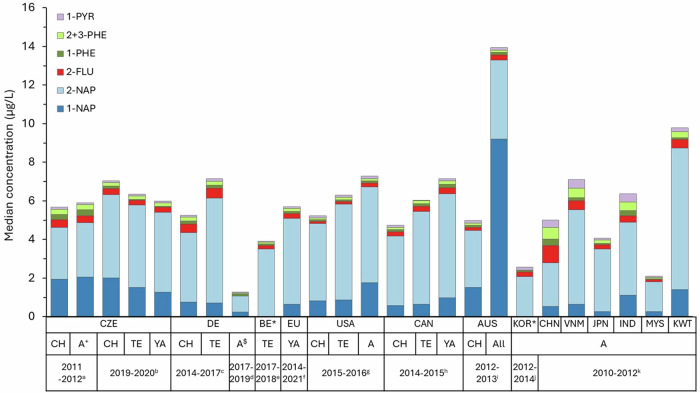
Profiles of selected OH-PAH metabolites for different countries/HBM survey. **a,** **b** our study, **c** [22], **d** [30], **e** [21], **f** [25], **g** [74], **h** [73], **i** [23], **j** [75], **k** [76]* 1-NAP was not measured in Belgium and Korea; + only women; All - age range 0–60 + ; ^$^ young adults, non-smokers, GM for the whole Australian population was used for comparison of adults as individual GM 1-OH-NAP strata in adults differed by up to two orders of magnitude.

The only metabolite for which we observed an increase between sampling periods in our study was 2-OH-NAP in non-smokers. Higher concentrations of 2-OH-NAP to 1-OH-NAP were observed in most countries (except Australia) (Fig. 4). Jung et al., 2014 [29] reported that the main exposure route of naphthalene is inhalation in the non-occupational environment and that increased urinary 2-OH-NAP could be due to increased emissions from the outdoor/indoor air. Burkhardt et al., 2023 [30] discussed that while the decrease in 1-OH-NAP may be partly explained by reduced exposure to second-hand tobacco smoke, the cause of the increasing 2-OH-NAP levels may be a combination of factors, such as different pathways of naphthalene metabolism [47], and an increase in exposure to other compounds that may be metabolised to 2-OH-NAP. Naphthalene is not just a combustion byproduct but is used in plastics, dyes, and pesticides and as a moth repellent in air fresheners and flooring [65]. Some studies suggest that the change in the 1-OH-NAP/2-OH-NAP ratio could be due to the carbaryl ban, but this is contested by others [66] and it does not support our findings since carbaryl was banned in the EU in 2007 [67]. It seems more probable that reduced overall naphthalene exposure may have altered metabolism, shifting the ratio in favour of 2-OH-NAP. If individuals are continuously exposed to higher levels of naphthalene, more 1-OH-NAP is produced during metabolism, resulting in an increased 1-OH-NAP/2-OH-NAP ratio [66]. Scherer et at. (2022) [47] found that smokers show higher 1-OH-NAP excretion, possibly due to enzyme induction from increased naphthalene exposure. The same trend is also evident in DEMOCOPHES-CZ smokers compared to non-smokers in our study, the median ratio of 1-OH-NAP/2-OH-NAP concentrations is 1.3 in smokers, while 0.7 in non-smokers. This is also supported by Zhu et al. [68], who observed individual variation in the ratio, and other studies noting shifts in this ratio to 1-OH-NAP in polluted areas [23, 69] or during occupational exposure [70, 71]. Another explanation could be the influence of emerging environmental chemicals that affect naphthalene metabolism, but the specific chemicals remain unidentified [65, 72].

The correlation between 1-OH-NAP and 2-OH-NAP is r_s_ = 0.74–0.85 in CELSPAC children, teenagers, and adults (Fig. S1c,d), indicating a similar exposure source and shift in 1-OH-NAP/2-OH-NAP ratio in smokers in our study, supporting the hypothesis of different pathways of naphthalene metabolism. However, further studies are needed to clarify the behaviour and sources of urinary OH-PAHs and to determine whether the apparent increase in urinary 2-OH-NAP in non-smokers and children may also occur under reduced exposure conditions due to naphthalene metabolism.

### Comparison with other countries

For comparison of urinary OH-PAH concentration with other countries, data from representative HBM surveys such as the Canadian Health Measures Survey (CHMS) [73], the US National Health and Nutrition Examination Survey (NHANES) [74], the HBM4EU Aligned Studies (including FR; PL; HR; PT; LU; DK; CZ; CH; DE; IS, not all metabolites were measured in all countries) [25] the Australian Population Survey [23] and Korean National Environmental Health Survey (KoNEHS) [75], and other data from studies with same age categories (children, adolescents and adults) and sampling years (2010–2021) corresponding to our study were selected. From NHANES and CHMS, data from the most recent available sampling cycles for OH-PAHs 2015–2016 and 2014–2015 were used. Comparisons of median/geometric means of individual metabolite concentrations are presented in Table S11.

There is uncertainty in comparing concentrations and ratios of OH-PAHs from different HBM surveys/studies due to differences in sampling year and season, detection and quantification limits, and analytical methods, but our aim was to show the pattern in exposure compared to representative HBM surveys. In particular, comparisons with Asian countries from the study by [76] should be taken with caution due to the very low sample size (ranging from 23 to 84 for each country), which cannot represent the general population of e.g. India or China.

The most commonly measured OH-PAHs were used to compare the OH-PAH profile among countries (Fig. 4). OH-NAPs are most abundant in all countries and age categories, with a higher proportion of 2-OH-NAP to 1-OH-NAP (except in the general Australian population), as was discussed above. A higher proportion of 2-OH-FLUO and 1,2,3-PHEs and 1-OH-PYR were found in some Asian countries such as China, India, Vietnam, and Kuwait (sampling from 2010–2011) compared to Europe, USA, Canada, and Australia; this pattern has been partially reported in Thai et al., [23]. A higher proportion of 1-OH-PYR is also seen in CZ-DEMOCOPHES mothers and children and German children and teenagers (Figs. 1b and 4).

The median concentrations of 1-OH-NAP in CELSPAC children and teenagers are twice as high as those in GerES V, CHMS, and US NHANES, but similar to Australian teenagers. In CELSPAC young adults, median concentrations of 1-OH-NAP are twice as high as HBM4EU, but similar to CHMS and US NHANES. The several-fold higher median 1-OH-NAP concentrations measured in the Australian survey were higher not only compared to the Czech population but also to all other countries [23]. CELSPAC young adults have 2-OH-NAP median concentrations similar to HBM4EU, Australian, CHMS, KoNEHS, and US NHANES, but four times higher than German non-smokers. Median concentrations of fluoranthene, phenanthrene, and pyrene metabolites are generally low in all countries (Table S11) and similar to the results from the Czech population.

## Conclusion

The study shows a decline in PAH metabolites in the Czech population over a ten-year period, in the context of legislative measures such as the smoking ban and the Air Protection Act. The ban on smoking in public places, along with the expansion of smoke-free alternatives, particularly affected smokers, whose urinary levels of all PAH metabolites notably decreased, some metabolites even by several-fold. Smaller reductions of 10–51% in PAH metabolite levels observed in non-smokers may be related to the smoking ban and the reduction in passive smoking, as well as the documented decline in PAH air pollution, supported by adopted legislative measures such as the replacement of domestic boilers, transition to natural gas, and emission-free heating. Significant exposure factors found in our study, such as fish and offal consumption and seasonality, explained only 4–7% of the variability in urinary PAH metabolites. The increase in 2-OH-NAP among nonsmokers and children, along with the different 1-OH-NAP/2-OH-NAP ratio warrants further investigation—particularly regarding naphthalene sources and metabolism. These findings underscore the importance of regulatory interventions in reducing PAH exposure and highlight the need for regular evaluation of the effectiveness of these measures and monitoring changes in population exposure.

## Supplementary information

Supplementary Material

Reporting Checklist

## Data Availability

Per RECETOX Research Infrastructure Board approval, the data that support the findings of this study are restricted for transmission to those outside the primary investigative team. Data sharing with investigators outside the team requires IRB approval. Requests may be submitted to Petra Růžičková, Ph.D. (petra.ruzickova@recetox.muni.cz).
