# Supplementary material for: Changing pattern of exposure to polycyclic aromatic hydrocarbons over time in the Central European population
Source: J Expo Sci Environ Epidemiol. 2025 Jul 17;36(1):184–98. doi: 10.1038/s41370-025-00793-z (PMC12795755; doi:10.1038/s41370-025-00793-z)
Supplement: Supplementary file 1 — Supplementary Material [file 41370_2025_793_MOESM1_ESM.pdf]

Supplementary Material:

Changing pattern of exposure to polycyclic aromatic hydrocarbons over time in the Central European population

Soňa Smetanová[1a], Akrem Jbebli[1a], Jiří Kohoutek[1], Vladimíra Puklová[2], Milena Černá[2,3], Andrea Krsková[2], Martin Zvonař[1,4], Zdenko Reguli[2], Lenka Andrýsková[1], Pavel Piler[1], Petra Přibylová[1], Jana Klánová[1], Elliott J. Price[1*], Klára Komprdová[1*]

[1] RECETOX, Faculty of Science, Masaryk University, Kotlarska 2, Brno, Czech Republic

[2] National Institute of Public Health, Šrobárova 48, Prague 10 100 42, Czech Republic

[3] Charles University, Third Faculty of Medicine, Prague, Czech Republic

[4] Faculty of Sports Studies, Masaryk University, Kamenice 753/5, Brno, Czech Republic

[a] These authors contributed equally.

*Correspondence addressed to: Klára Komprdová (klara.komprdova@recetox.muni.cz) and/or Elliott J. Price (elliott.price@recetox.muni.cz)

Supplementary method

<u>Urinary OH-PAH analysis</u>

Urine spot samples were stored at -80°C before analysis. Samples were defrosted, stabilized at ambient temperature (~20 °C), and homogenized with a Wizard Advanced IR vortex mixer (Velp Scientifica, USA). Sample aliquots (500 µL) were transferred to a 96-well sample collection plate with 2 mL square wells (Waters, Prague), 500 µL hydrolysis solution containing β-glucuronidase (1kU/mL) and isotopically labelled standards (each at 1 µg/ mL) was added and mixed by aspiration. The well plate was closed with a foil cover, and samples were incubated for 120 min at 55 °C on a thermoblock.

Following incubation, an Oasis HLB 96-well SPE plate (60mg; Waters) was conditioned with 1 mL methanol (LC-MS grade; Biosolve), then 1 mL ultrapure (type I) water (Milli-Q; Millipore). Samples were transferred to the conditioned wells and loaded without the aid of a vacuum. Each well was washed with 1 mL 40% methanol under a mild vacuum, and the SPE plate was dried for 30 s. Extracts were eluted via the addition of 1.5 mL acetone (Pesticide Residue Analysis grade; T. Baker) under a slight vacuum and eluates collected into a fresh 96-well samples collection plate containing 10 µL DMSO (ReagentPlus grade, Sigma) per well. Extracts were concentrated to 10 µL under nitrogen whilst incubated at 55 °C. Subsequently, 100 µL 50% methanol was added, and samples were vortexed and stored at 4 °C prior to further analysis.

OH-PAHs were analyzed via liquid chromatography tandem mass spectrometry (LC-MS/MS) using an Agilent 1200 series liquid chromatography (HPLC) system coupled to an AB Sciex Qtrap 5500 mass spectrometer. Extracts (5 µL) were separated using a Waters Acquity BEH C18 analytical column (100 x 2.1 mm, 1.7 µm particle size) equipped with Acquity BEH C18 VanGuard pre-column (5 x 2.1 mm, 1.7 µm particle size). The column compartment and autosampler were maintained at 30 °C and 10 °C, respectively. Water with 0.1 mM ammonium fluoride (NH_4F) (eluent A) and methanol with 0.1 mM NH_4F (eluent B) were used as mobile phases. The flow rate was 0.3 mL/min with a linear gradient from 50% B to 100% B over 7 min and held for 3 min before returning to initial conditions.

Multiple reaction monitoring (MRM) experiments were performed in negative electrospray ionization (ESI) mode at 450°C with N_2 as a nebulizer gas and a capillary voltage of -4kV; further details are provided in Table S1. Quantification of OH-PAHs was via stable-isotope dilution method to respective labelled standards; with 1- hydroxynaphthalene reported relative to 2-hydroxynaphthalene ($^{13}C6$); 9-hydroxyphenanthrene relative to 1-hydroxyphenanthrene ($^{13}C4$) and 3-OH-benzo[a]pyrene with respect to 1-hydroxypyrene ($^{13}C6$). The linear quantification range (MRM mode) was 0.1-100 µg/L urine, with minimum quantification limits (MQL) ranging from 0.018 to 0.2 µg/L urine for respective analytes (MQL calculated as 10* the standard deviation (SD) of the blank sample set concentration).

Each batch of samples contained a blank matrix, SRM QC material, and in-house QC samples. QC samples were used to check the performance of the sample preparation method and instrumental drift (system suitability). Matrix blank was subtracted for each analyte per batch to eliminate the background and noise contribution to the analyte concentration. No batch effect was observed, so no batch correction was applied. Quality control is ensured by successful participation in the HBM4EU quality assurance (QA)/QC programme (1) with four rounds of Inter-laboratory Comparison Investigations (ICIs) and External Quality Assurance Schemes (EQUASs) (2).

<u>Urinary creatinine analysis</u>

Creatinine levels in urine were determined by LC-MS/MS method using a modified procedure of Dereziński *et al*. (3). In brief, 10 µL urine was added to 490 µL of 2% formic acid in a 1.5mL Eppendorf microtube. Following addition, the sample was vortexed and centrifuged for 2 minutes at 10,000g. Subsequently, 5 µL of supernatant was removed to clean a 2mL glass autosampler vial with the addition of 995 uL of creatinine-d_3 solution (c=20ng/mL). The sample was thoroughly mixed using vortex (2x 5 s). Blank samples comprised 10 µL type 1 ultrapure (Milli-Q) water, whilst Seronorm™ Trace Elements Urine L-1 and L-2 certified reference materials were used as QA/QC materials to verify the performance of the method.

An Agilent HPLC 1200 series coupled to an Agilent 6495A triple quadrupole MS was used for analysis. A Phenomenex LUNA C_{18} column (100 x 2 mm, 3 µm particle size) equipped with a Phenomenex SecureGuard C_{18} pre-column was maintained at 30 °C and the mobile phase consisted of 0.1% formic acid in MilliQ water/acetonitrile mixture (80/20, v/v), with isocratic elution at a flow rate of 0.25 mL/min. The injection volume was 5 µL.

Analyte was detected in positive electrospray ionization mode. Nitrogen was used as a nebulizer and desolvation gas (350 degC., 14L/min), and the capillary voltage was 3.5 kV. Multiple reaction monitoring was used for acquisition, recording the signal from m/z 114 → 44 for quantification and 114 → 85.8 for qualification. The method quantitation limit (S/N 10:1, peak-to-peak method) was 0.84 ng/mL, and the linear quantification range was 1-300 mg/dL, with limits of detection and quantification of 0.25 and 0.84 mg/dL, respectively.

<u>Age and sex differences</u>

In this study, both the DEMOCOPHES-CZ and CELSPAC cohorts had higher concentrations of measured OH-PAHs in children compared with adult nonsmokers, except for 4-OH-PHE and 9-OH-PHE, which were lower. The higher OH-PAH concentrations in the children's urine may be explained by their different behavior patterns (playing on the ground, higher hand-to-mouth interaction, and breathing rate) and higher dietary intake in relation to body weight (4,5). Differences among age categories can be confounded by smoking and other factors that tend to vary with age. This can lead to different trends reported in existing studies. Thai et al., 2016 (6) reported a lower concentration of all measured OH-PAHs in children than in teenagers or

adults, however both smokers and non-smokers were included in the study. In the Korean National Environmental Health Survey (7), preschool children had significantly higher concentrations of 2-OH-NAP and 2-OH-FLUO than other age groups (smokers were included). Metanalysis made by Huang et al., 2019 (8) and results from NHANES study (9) also pointed to differences in OH-PAHs in children and adolescents with significantly lower 1-OH-PYR (both studies) and 1-PHEN, 2-PHEN, 3-PHEN, 4-PHEN (NHANES study) concentration in children.

The level of creatinine in the urine is age- and sex-dependent (10). Increasing creatinine concentration with age and significantly higher creatinine in adult males than females (KW, $p<0.05$) were also found in CELSPAC cohort. The differences in sex and age were tested both for adjusted and non-adjusted metabolite concentration in our study with similar results, so we do not expect a significant influence of standardization. However, the effect of creatinine adjustment should be considered when interpreting results.

Air concentrations

The PAH levels (parent compounds of metabolites measured in urine) were checked in the three different cities where the participants live, namely Brno and surrounding areas for CELSPAC (school children, teenagers and young adults) and Liberec and Prague for DEMOCOPHES-CZ (mother and their children), and at Czech national station for monitoring background emissions in Košetice from Global Environmental Assessment and Information System (GENASIS) (11). The values reported as annual median values (measured by passive sampling on PUF) are available for EMEP (European Monitoring and Evaluation Program) stations in Prague (Prague 4 – Libuš) and Košetice, for CHMI station in Brno-Líšeň and for MONET station in Liberec (Ještěd). There is a significant decrease in the annual median concentrations of fluorene, phenanthrene, and naphthalene within the period 2011-2020 measured at the EMEP station in Košetice. Data from the monitoring stations in Brno, Prague, and Liberec confirmed the trends measured in Košetice. Also, it must be noted that pyrene concentrations did not decrease in the air during the monitoring period. The conclusions regarding trends of PAHs in the air were taken directly from the Global Environmental Assessment and Information System (GENASIS) (11), where PAH concentrations are already statistically processed. To check the information, visit the website https://data.genasis.cz/#/outdoor/spatial-distribution, select the "Time Series Analysis" option, and enter the search filters correctly. For further information, see Table S10.

Table S1 Transitions and parameters for targeted detection of OH-PAHs.

Analyte	Rt[1]	Q1	Q3	DP[2]	EP[3]	CE[4]	CXP[5]
**1-OH-NAP_1**	2.7	143	115	-120	-3	-34	-12
**1-OH-NAP_2**	2.7	143	41	-260	-3	-34	-12
**2-OH-NAP_1**	2.6	143	115	-120	-3	-34	-12
**2-OH-NAP_2**	2.6	143	41	-260	-3	-34	-12
**2-OH-FLUO**	5	181	180	-100	-3	-34	-12
**3-OH-FLUO**	4.9	181	180	-100	-3	-26	-12
**1-OH-PHE**	7.2	193	165	-100	-3	-38	-12
**2-OH-PHE**	6.2	193	165	-150	-3	-41	-12
**4-OH-PHE**	7.5	193	165		-3	-38	-12
**9-OH-PHE**	6.9	193	165		-3	-38	-12
**1-OH-PYR**	9.1	217	189	-100	-3	-45	-12
**3-OH-BaP**	9.9	267	239	-105	-10	-50	-25
**$^{13}C_6$-2-OH-NAP**	2.6	149	121	-105	-10	-32	-13
**$^{13}C_6$-2-OH-FLUO**	5	187	186	-95	-10	-32	-27
**$^{13}C_6$-3-OH-FLUO**	4.9	187	159	-50	-10	-32	-9
**$^{13}C_4$-1-OH-PHE**	7.2	197	168	-100	-3	-36	-12
**$^{13}C_6$-2-OH-PHE**	6.2	199	171	-150	-3	-41	-12
**$^{13}C_4$-4-OH-PHE**	7.5	197	168	-100	-3	-36	-12
**$^{13}C_6$-1-OH-PYR**	9.1	223	195	-100	-3	-36	-12

[1]Retention time

[2]Declustering potential

[3]Entrance potential

[4]Collision energy

[5]Collision cell exit potential

Table S2 Percentage of samples above limit of detection (LOD) for each cohort.

**Cohort**			**DEMOCOPHES-CZ**		**CELSPAC**		
			MOTHERS	CHILDREN	CHILDREN	TEENS	YOUNG ADULTS
**Sampling year**			2011-2012		2019-2020		
**N samples**			116	119	195	299	315
**Metabolite**	**IUPAC ; InChI**	**LOD**	**Percentage of samples above LOD**				

1-OH-NAP	naphthalen-1-ol ; 1S/C10H8O/c11-10-7-3-5-8-4-1-2-6-9(8)10/h1-7,11H	0.3	96.6	99.2	98.5	95.3	93
2-OH-NAP	naphthalen-2-ol ; 1S/C10H8O/c11-10-6-5-8-3-1-2-4-9(8)7-10/h1-7,11H	0.37	98.3	100	100	99.7	98.7
2-OH-FLUO	9H-fluoren-2-ol ; 1S/C13H10O/c14-11-5-6-13-10(8-11)7-9-3-1-2-4-12(9)13/h1-6,8,14H,7H2	0.03	100	100	100	98	99.7
3-OH-FLUO	9H-fluoren-3-ol ; 1S/C13H10O/c14-11-6-5-10-7-9-3-1-2-4-12(9)13(10)8-11/h1-6,8,14H,7H2	0.02	92.2	97.5	92.3	90	81.9
1-OH-PHE	phenanthren-1-ol ; S/C14H10O/c15-14-7-3-6-12-11-5-2-1-4-10(11)8-9-13(12)14/h1-9,15H	0.03	99.1	100	99.5	68.1	67.9
2/3-OH-PHE	phenanthren-2-ol ; 1S/C14H10O/c15-12-7-8-14-11(9-12)6-5-10-3-1-2-4-13(10)14/h1-9,15H / phenanthren-3-ol ; 1S/C14H10O/c15-12-8-7-11-6-5-10-3-1-2-4-13(10)14(11)9-12/h1-9,15H	0.07	95.7	100	91.3	90.4	90.8
4-OH-PHE	phenanthren-4-ol ; 1S/C14H10O/c15-13-7-3-5-11-9-8-10-4-1-2-6-12(10)14(11)13/h1-9,15H	0.02	91.4	95.8	66.7	92.4	70.8
9-OH-PHE	phenanthren-9-ol ; 1S/C14H10O/c15-14-9-10-5-1-2-6-11(10)12-7-3-4-8-13(12)14/h1-9,15H	0.06	87.1	82.4	9.2	45.2	50.8
1-OH-PYR	pyren-1-ol ; 1S/C16H10O/c17-14-9-7-12-5-4-10-2-1-3-11-6-8-13(14)16(12)15(10)11/h1-9,17H	0.05	75.9	95	77.9	89	81.6
3-OH-BaP	benzo[a]pyren-3-ol ; 1S/C20H12O/c21-18-10-7-12-5-8-16-15-4-2-1-3-13(15)11-14-6-9-17(18)19(12)20(14)16/h1-11,21H	0.08	11.2	7.6	0	1	0

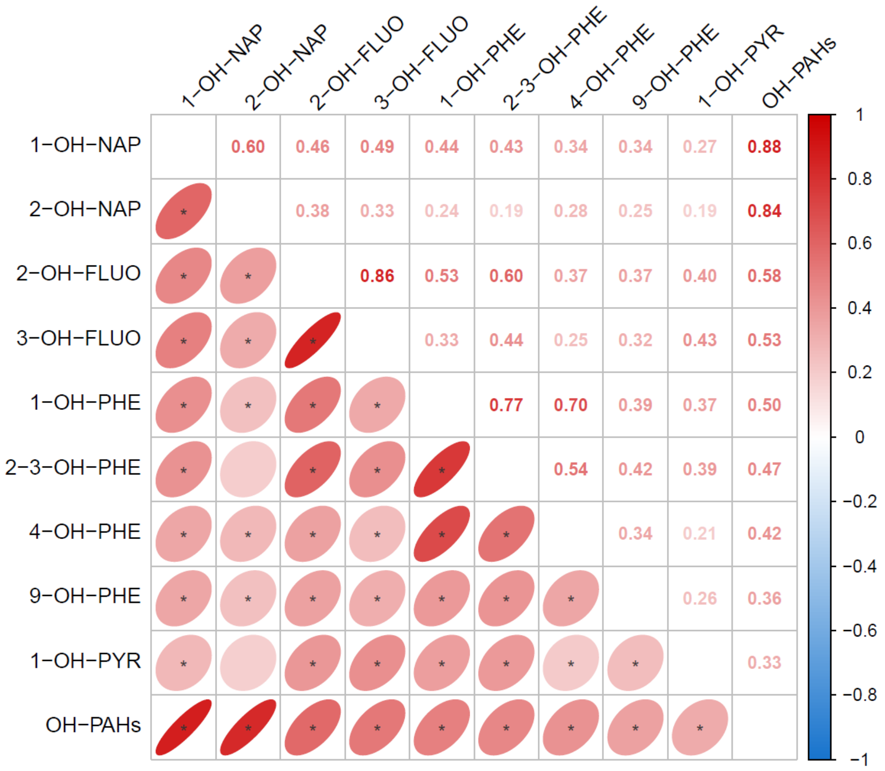

a) DEMOCOPHES mothers

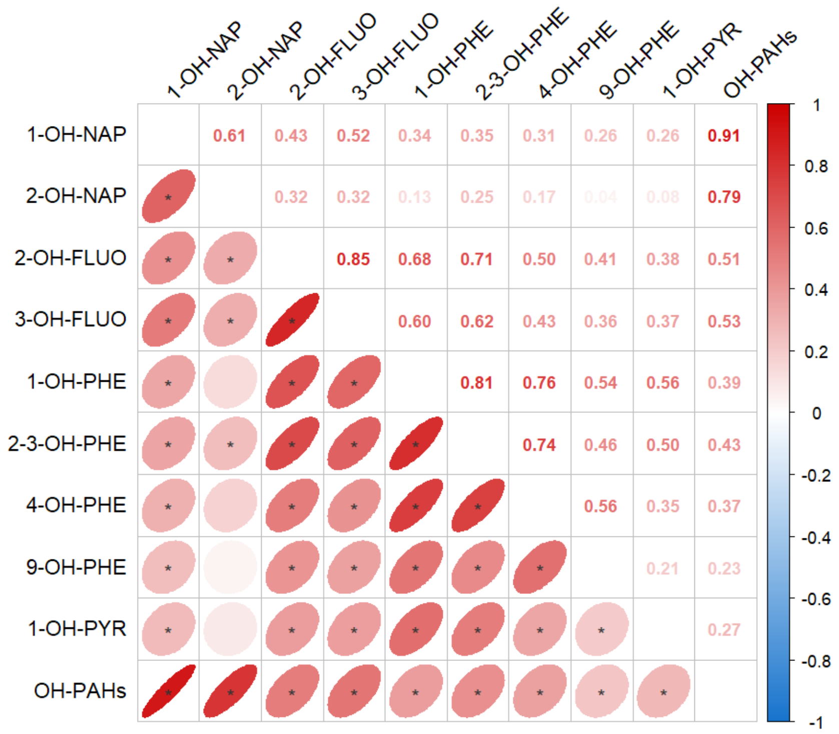

b) DEMOCOPHES children

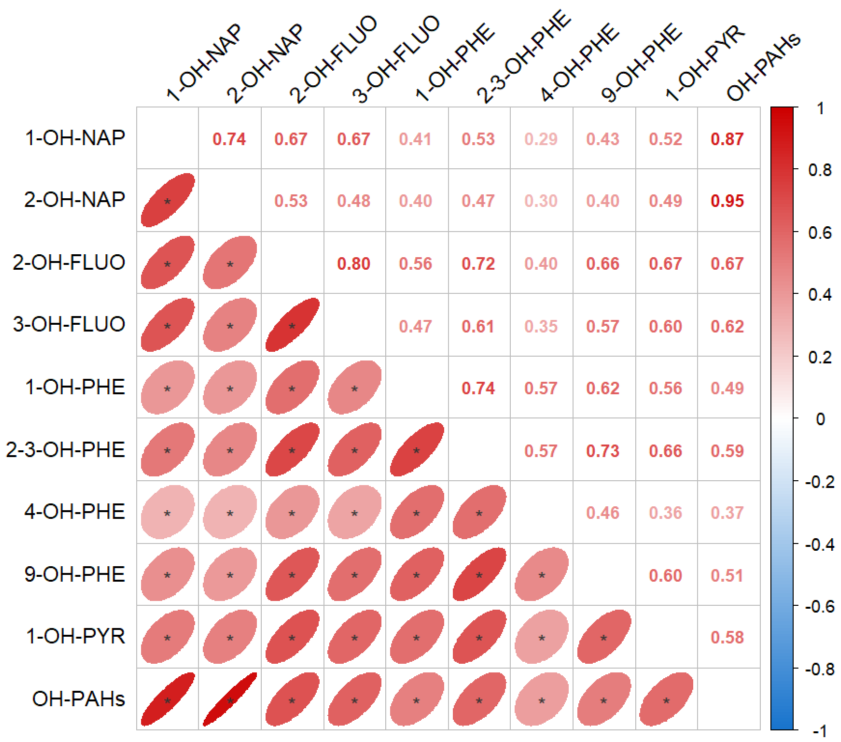

c) CELSPAC teenagers and young adults

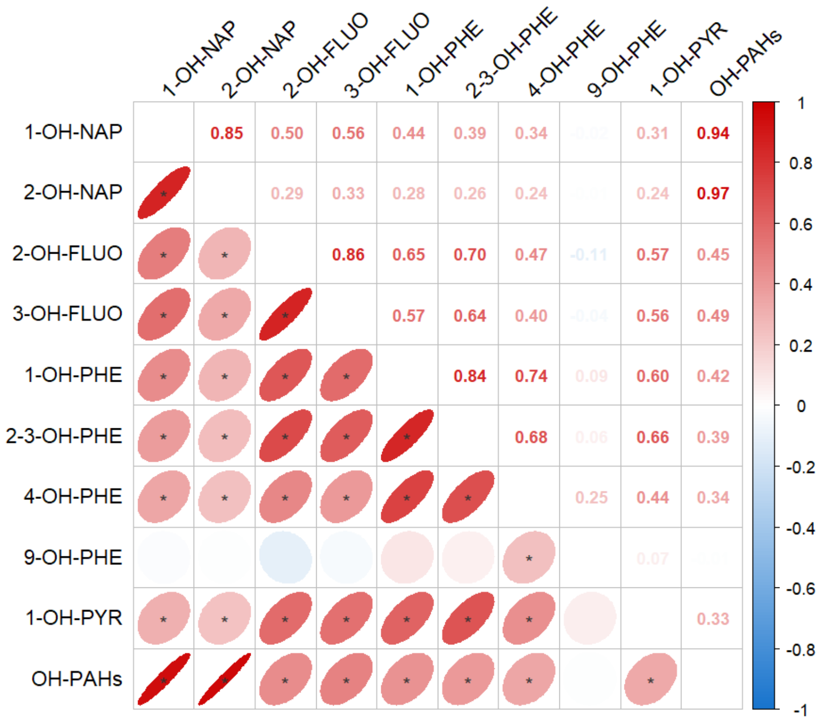

d) CELSPAC children; low number of samples above LOD for 9-OH-PHE

Figure S1 a-e Spearman correlations between PAH metabolites. All correlations are statistically significant. Correlations were performed for each cohort and age group separately; smokers were excluded from the analysis.

Table S3 Descriptive statistics of the non-adjusted urinary concentrations of PAH metabolites recorded for the three cohorts. Values below LOD were replaced by LOD/√2.

Cohort study/Population	Biomarker	Min	5P	25P	Median	GM	75P	95P	Max
CHILDREN (DEMOCOPHES-CZ), N=119	**1-OH-NAP**	<LOD	0.614	1.226	1.933	2.358	3.995	11.059	219.743
	**2-OH-NAP**	0.458	0.695	1.572	2.701	2.657	4.261	9.682	15.597
	**2-OH-FLUO**	0.090	0.133	0.279	0.402	0.416	0.687	1.128	4.264
	**3-OH-FLUO**	<LOD	0.021	0.061	0.102	0.097	0.166	0.279	0.636
	**1-OH-PHE**	0.039	0.105	0.181	0.267	0.268	0.378	0.638	3.699
	**2-3-OH-PHE**	0.070	0.110	0.177	0.254	0.269	0.378	0.757	2.992
	**4-OH-PHE**	<LOD	0.021	0.038	0.053	0.059	0.091	0.168	0.930
	**9-OH-PHE**	<LOD	<LOD	0.073	0.122	0.121	0.196	0.407	1.026
	**1-OH-PYR**	<LOD	<LOD	0.084	0.131	0.127	0.187	0.347	1.084
	**3-OH-BaP**	<LOD	<LOD	<LOD	<LOD	<LOD	<LOD	0.103	0.404
	**ΣOH-NAP**	0.777	1.512	3.080	4.900	5.430	8.529	20.237	222.284
	**ΣOH-FLUO**	0.110	0.159	0.347	0.504	0.518	0.845	1.464	4.698
	**ΣOH-PHE**	0.193	0.323	0.506	0.725	0.750	1.157	2.010	7.913
MOTHERS (DEMOCOPHES-CZ), N=116	**1-OH-NAP**	<LOD	0.386	1.148	2.054	2.454	5.126	24.186	42.157
	**2-OH-NAP**	<LOD	0.669	1.675	2.816	2.996	5.210	17.913	82.297
	**2-OH-FLUO**	0.042	0.109	0.207	0.349	0.372	0.629	1.936	3.208
	**3-OH-FLUO**	<LOD	<LOD	0.038	0.065	0.074	0.134	0.633	1.106
	**1-OH-PHE**	<LOD	0.084	0.170	0.321	0.291	0.477	0.955	1.452
	**2-3-OH-PHE**	<LOD	0.074	0.150	0.257	0.249	0.406	0.781	1.624
	**4-OH-PHE**	<LOD	<LOD	0.038	0.061	0.063	0.113	0.261	0.345
	**9-OH-PHE**	<LOD	<LOD	0.100	0.156	0.161	0.236	0.791	4.520
	**1-OH-PYR**	<LOD	<LOD	0.055	0.095	0.090	0.152	0.294	0.707
	**3-OH-BaP**	<LOD	<LOD	<LOD	<LOD	<LOD	<LOD	0.108	0.198
	**ΣOH-NAP**	0.474	1.012	2.910	4.958	5.778	11.147	39.889	98.973
	**ΣOH-FLUO**	0.056	0.127	0.256	0.430	0.451	0.773	2.368	3.973
	**ΣOH-PHE**	0.138	0.246	0.513	0.819	0.818	1.300	2.553	6.175
YOUNG ADULTS (CELSPAC), N=315	**1-OH-NAP**	<LOD	<LOD	0.633	1.270	1.446	3.015	11.890	63.800
	**2-OH-NAP**	<LOD	0.845	2.155	4.130	4.104	7.400	20.400	68.000
	**2-OH-FLUO**	<LOD	0.073	0.153	0.270	0.294	0.490	1.530	24.400
	**3-OH-FLUO**	<LOD	<LOD	0.030	0.050	0.059	0.110	0.505	3.900
	**1-OH-PHE**	<LOD	<LOD	<LOD	0.040	0.048	0.070	0.252	7.260
	**2-3-OH-PHE**	<LOD	<LOD	0.110	0.180	0.201	0.350	0.744	11.800
	**4-OH-PHE**	<LOD	<LOD	<LOD	0.090	0.069	0.160	0.598	2.960
	**9-OH-PHE**	<LOD	<LOD	<LOD	0.210	0.135	0.368	0.780	23.000
	**1-OH-PYR**	<LOD	<LOD	0.060	0.090	0.098	0.160	0.313	11.900
	**3-OH-BaP**	<LOD	<LOD	<LOD	<LOD	<LOD	<LOD	<LOD	<LOD
	**ΣOH-NAP**	0.474	1.351	3.090	5.690	5.920	11.330	31.868	102.800
	**ΣOH-FLUO**	0.035	0.087	0.190	0.340	0.360	0.598	2.090	28.300
	**ΣOH-PHE**	0.127	0.136	0.239	0.512	0.523	0.949	2.027	45.020
[illegible]	**1-OH-NAP**	<LOD	0.305	0.853	1.520	1.652	3.100	9.856	45.400

	**2-OH-NAP**	<LOD	0.970	2.595	4.270	4.608	8.650	22.200	114.000
	**2-OH-FLUO**	<LOD	0.060	0.150	0.250	0.253	0.430	1.117	5.800
	**3-OH-FLUO**	<LOD	<LOD	0.040	0.070	0.070	0.120	0.390	1.430
	**1-OH-PHE**	<LOD	<LOD	<LOD	0.040	0.042	0.060	0.151	0.910
	**2-3-OH-PHE**	<LOD	<LOD	0.100	0.170	0.173	0.270	0.680	2.880
	**4-OH-PHE**	<LOD	<LOD	0.040	0.070	0.062	0.100	0.190	0.900
	**9-OH-PHE**	<LOD	<LOD	<LOD	<LOD	0.115	0.318	0.751	5.250
	**1-OH-PYR**	<LOD	<LOD	0.060	0.100	0.104	0.160	0.332	1.700
	**3-OH-BaP**	<LOD	<LOD	<LOD	<LOD	<LOD	<LOD	<LOD	3.580
	**ΣOH-NAP**	0.474	1.481	3.715	5.980	6.580	12.170	30.839	139.200
	**ΣOH-FLUO**	0.035	0.094	0.190	0.300	0.332	0.558	1.493	7.210
	**ΣOH-PHE**	0.127	0.133	0.222	0.362	0.425	0.745	1.822	8.310
**SCHOOL CHILDREN (CELSPAC), N=195**	**1-OH-NAP**	<LOD	0.513	1.020	2.000	2.067	3.670	11.640	26.400
	**2-OH-NAP**	0.450	1.125	2.365	4.330	4.527	8.850	19.830	35.700
	**2-OH-FLUO**	0.050	0.093	0.170	0.310	0.322	0.540	1.369	4.740
	**3-OH-FLUO**	<LOD	<LOD	0.030	0.060	0.067	0.120	0.423	0.820
	**1-OH-PHE**	<LOD	0.040	0.090	0.140	0.154	0.270	0.530	1.460
	**2-3-OH-PHE**	<LOD	<LOD	0.100	0.160	0.181	0.310	0.676	1.630
	**4-OH-PHE**	<LOD	<LOD	<LOD	0.030	0.033	0.050	0.170	1.630
	**9-OH-PHE**	<LOD	<LOD	<LOD	<LOD	<LOD	<LOD	0.093	0.540
	**1-OH-PYR**	<LOD	<LOD	0.050	0.090	0.093	0.160	0.346	1.280
	**3-OH-BaP**	<LOD	<LOD	<LOD	<LOD	<LOD	<LOD	<LOD	<LOD
	**ΣOH-NAP**	0.662	1.658	3.785	6.700	6.746	13.285	30.718	58.400
	**ΣOH-FLUO**	0.064	0.111	0.203	0.370	0.395	0.685	1.699	5.540
	**ΣOH-PHE**	0.127	0.156	0.268	0.382	0.436	0.667	1.433	3.142

Table S4 Descriptive statistics of the specific gravity adjusted urinary concentrations of PAH metabolites recorded for the three cohorts. Values below LOD were replaced by LOD/√2.

**Cohort study/ Population**	**Analyte**	**SG adjusted**							
		Min	5P	25P	Median	GM	75P	95P	Max
**CELSPAC: YOUNG ADULTS**	1-OH-NAP	<LOD	<LOD	0.826	1.871	2.035	4.052	19.725	305.739
	2-OH-NAP	<LOD	1.157	2.621	5.659	5.779	12.376	30.524	209.739
	2-OH-FLUO	<LOD	0.100	0.207	0.382	0.414	0.704	2.461	28.566
	3-OH-FLUO	<LOD	<LOD	0.036	0.072	0.083	0.161	0.751	9.704
	1-OH-PHE	<LOD	<LOD	<LOD	0.060	0.068	0.107	0.425	8.500
	2-3-OH-PHE	<LOD	<LOD	0.153	0.269	0.283	0.500	1.496	17.061
	4-OH-PHE	<LOD	<LOD	<LOD	0.104	0.097	0.240	0.946	10.539
	9-OH-PHE	<LOD	<LOD	<LOD	0.216	0.190	0.528	1.357	26.927
	1-OH-PYR	<LOD	<LOD	0.075	0.126	0.138	0.231	0.542	13.932
	3-OH-BaP	<LOD	<LOD	<LOD	<LOD	<LOD	<LOD	<LOD	<LOD
	ΣOH-NAP	0.503	1.617	4.054	7.809	8.335	18.204	46.528	515.478
	ΣOH-FLUO	0.038	0.122	0.249	0.457	0.507	0.849	3.155	33.132
	ΣOH-PHE	0.096	0.171	0.354	0.676	0.736	1.456	3.965	52.706

**CELSPAC: TEENAGERS**	1-OH-NAP	<LOD	0.543	1.120	1.839	1.996	3.332	8.362	52.134
	2-OH-NAP	<LOD	1.642	3.136	5.058	5.568	9.424	24.485	160.941
	2-OH-FLUO	<LOD	0.091	0.194	0.289	0.306	0.453	1.199	5.858
	3-OH-FLUO	<LOD	<LOD	0.048	0.078	0.084	0.134	0.354	1.344
	1-OH-PHE	<LOD	<LOD	<LOD	0.045	0.051	0.071	0.205	1.045
	2-3-OH-PHE	<LOD	<LOD	0.137	0.196	0.209	0.303	0.612	3.307
	4-OH-PHE	<LOD	<LOD	0.051	0.080	0.075	0.110	0.185	0.881
	9-OH-PHE	<LOD	<LOD	<LOD	<LOD	0.138	0.299	0.740	7.119
	1-OH-PYR	<LOD	<LOD	0.087	0.118	0.126	0.181	0.314	1.952
	3-OH-BaP	<LOD	<LOD	<LOD	<LOD	<LOD	<LOD	<LOD	2.644
	ΣOH-NAP	0.628	2.423	4.779	7.403	7.950	13.322	32.765	196.518
	ΣOH-FLUO	0.046	0.131	0.247	0.363	0.401	0.576	1.396	7.085
	ΣOH-PHE	0.144	0.199	0.313	0.471	0.514	0.737	1.656	11.119
**CELSPAC: SCHOOL CHILDREN**	1-OH-NAP	<LOD	0.787	1.425	2.445	2.643	4.269	11.690	24.881
	2-OH-NAP	1.080	1.550	3.405	5.793	5.788	9.769	22.705	32.862
	2-OH-FLUO	0.075	0.155	0.255	0.383	0.412	0.587	1.539	3.817
	3-OH-FLUO	<LOD	<LOD	0.044	0.079	0.086	0.138	0.459	1.081
	1-OH-PHE	<LOD	0.070	0.135	0.185	0.197	0.280	0.757	1.184
	2-3-OH-PHE	<LOD	<LOD	0.148	0.210	0.232	0.340	0.728	1.754
	4-OH-PHE	<LOD	<LOD	<LOD	0.036	0.042	0.066	0.178	1.417
	9-OH-PHE	<LOD	<LOD	<LOD	<LOD	<LOD	<LOD	0.146	0.527
	1-OH-PYR	<LOD	<LOD	0.076	0.118	0.119	0.166	0.353	1.105
	3-OH-BaP	<LOD	<LOD	<LOD	<LOD	<LOD	<LOD	<LOD	<LOD
	ΣOH-NAP	1.581	2.282	4.920	8.478	8.624	14.154	35.257	55.817
	ΣOH-FLUO	0.090	0.191	0.307	0.462	0.505	0.747	2.083	4.462
	ΣOH-PHE	0.136	0.251	0.375	0.517	0.557	0.764	1.921	3.382

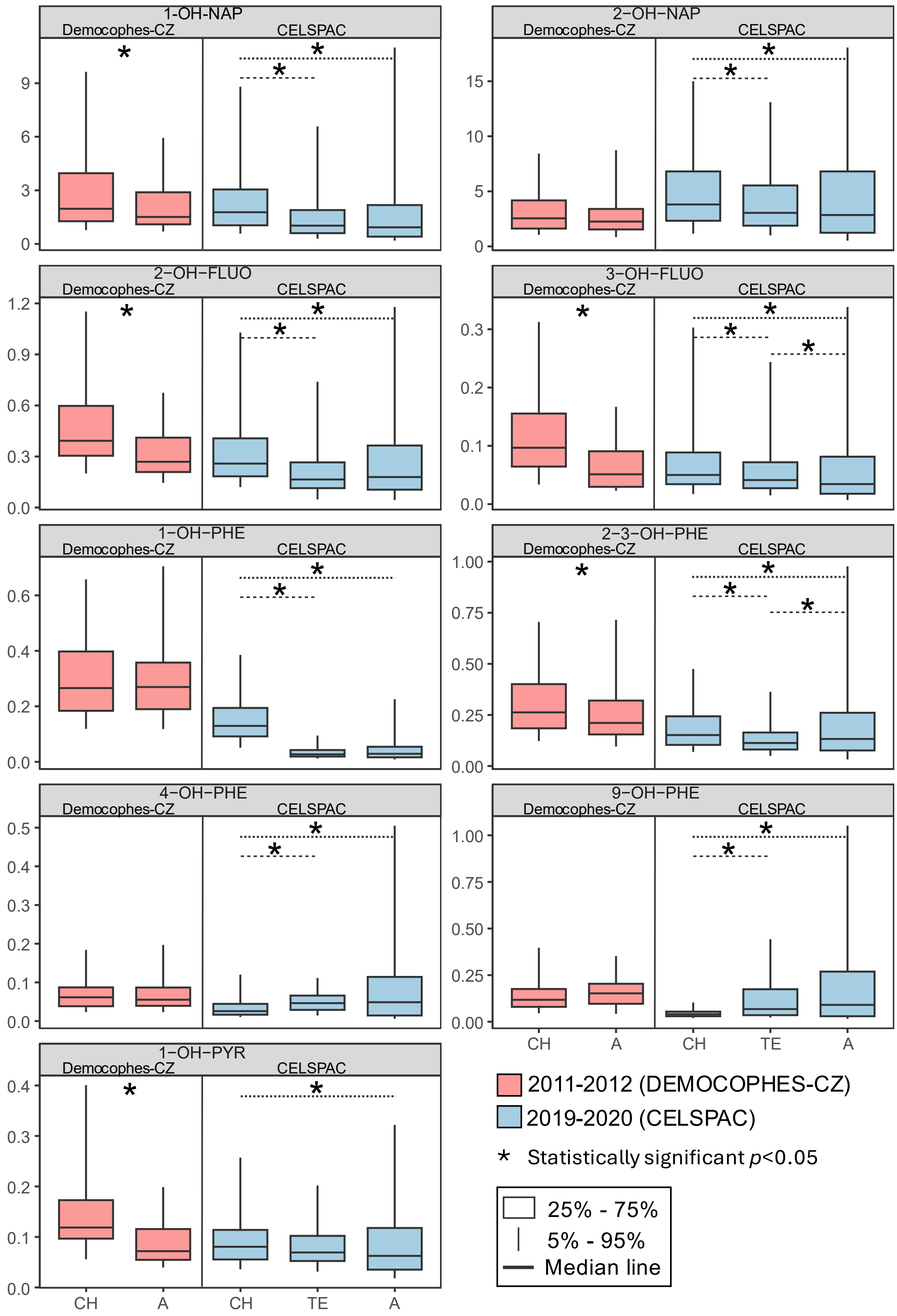

Figure S2 Comparison of OH-PAH concentrations (µg/g creatinine) between children and mothers in DEMOCOPHES-CZ and school children, teenagers and young adults in CELSPAC cohort. A statistically significant difference between groups is marked with an asterisk. Smokers were excluded.

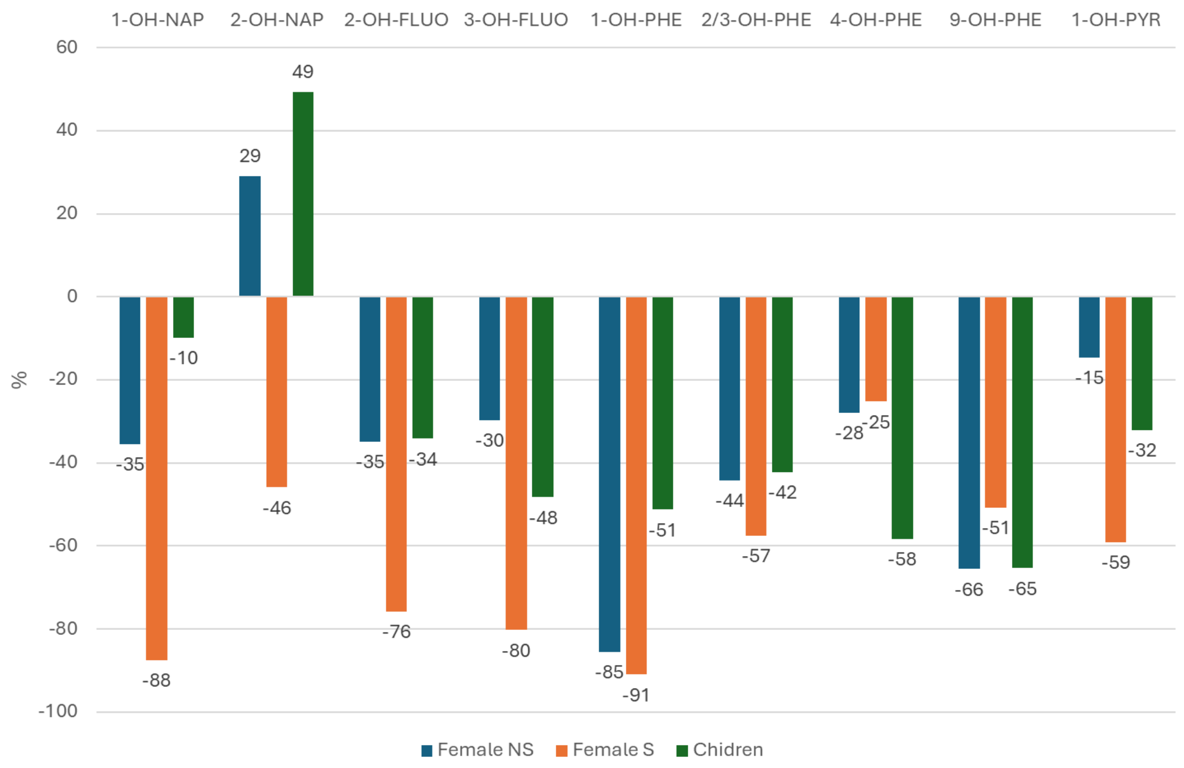

Figure S3 Percentage decrease/increase in the median creatinine-adjusted concentrations of PAH metabolites between sampling periods. NS-nonsmokers, S-smokers.

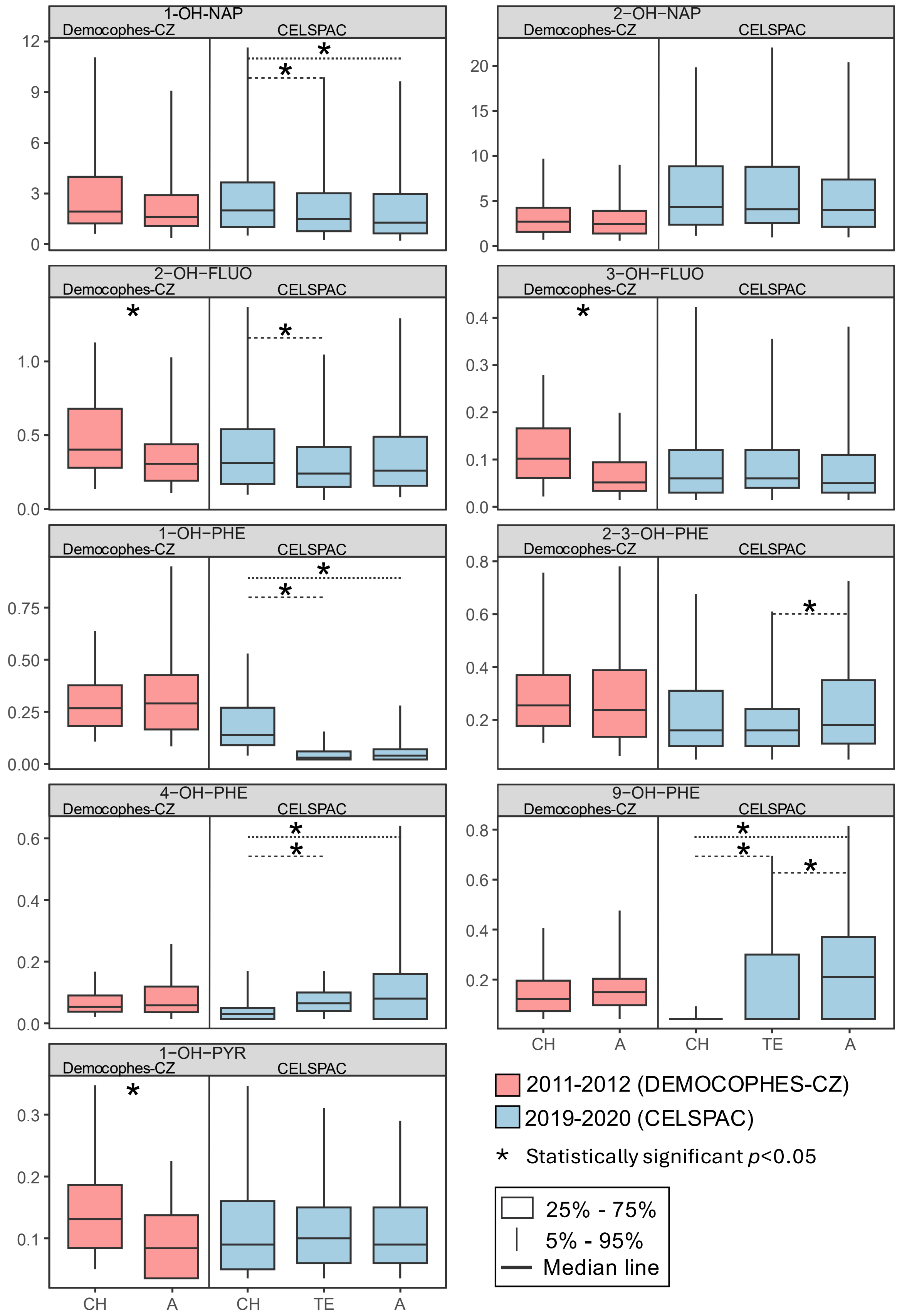

Figure S4 Comparison of OH-PAH concentrations (µg/g, non-adjusted) between children and mothers in DEMOCOPHES-CZ and school children, teenagers and young adults in CELSPAC cohort. A statistically significant difference between groups is marked with an asterisk. Smokers were excluded.

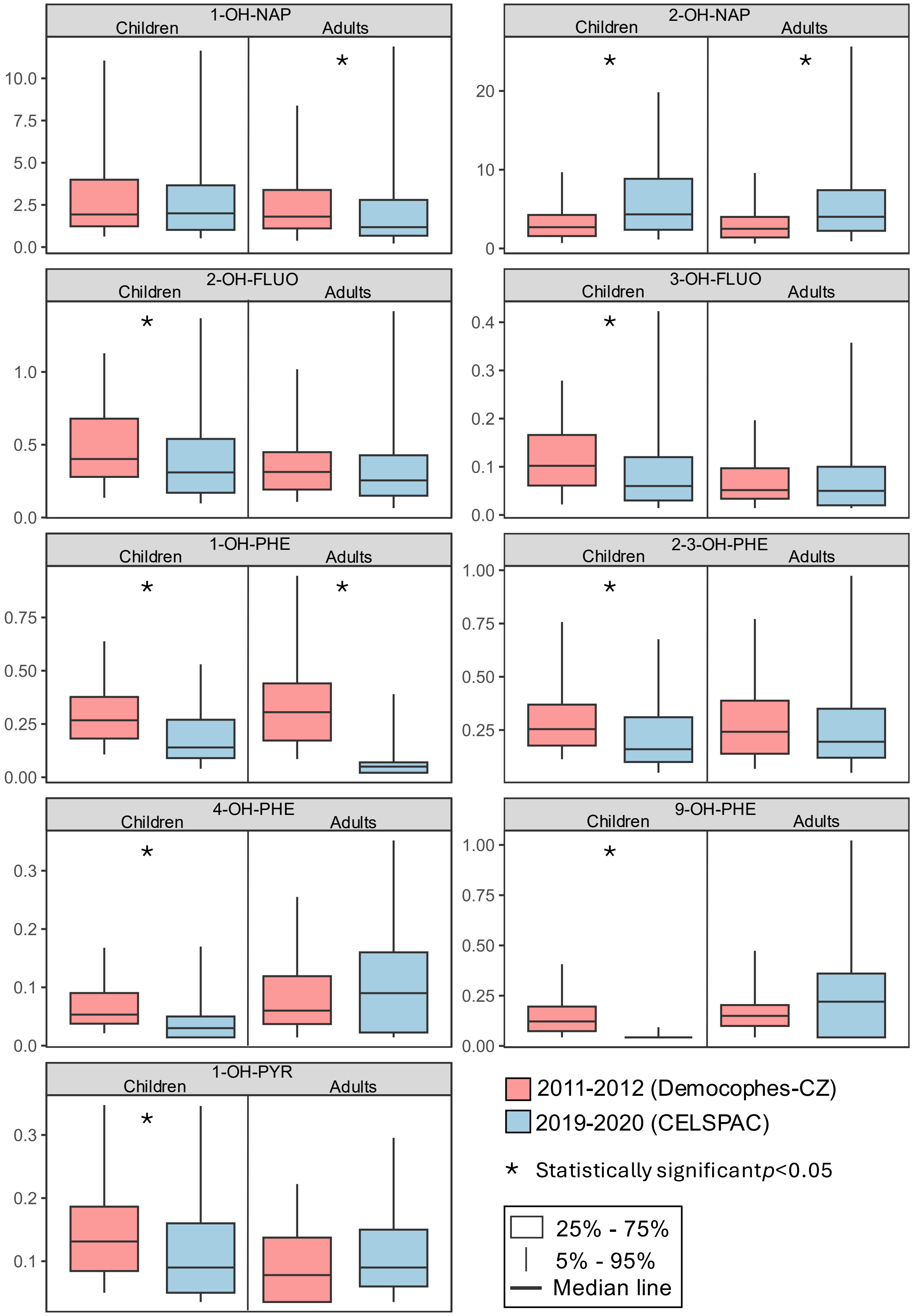

Figure S5 Comparison of OH-PAH non-adjusted metabolite concentrations (µg/L) between DEMOCOPHES-CZ and CELSPAC women (adults) and between DEMOCOPHES-CZ and CELSPAC children in two sampling periods. Smokers were excluded.

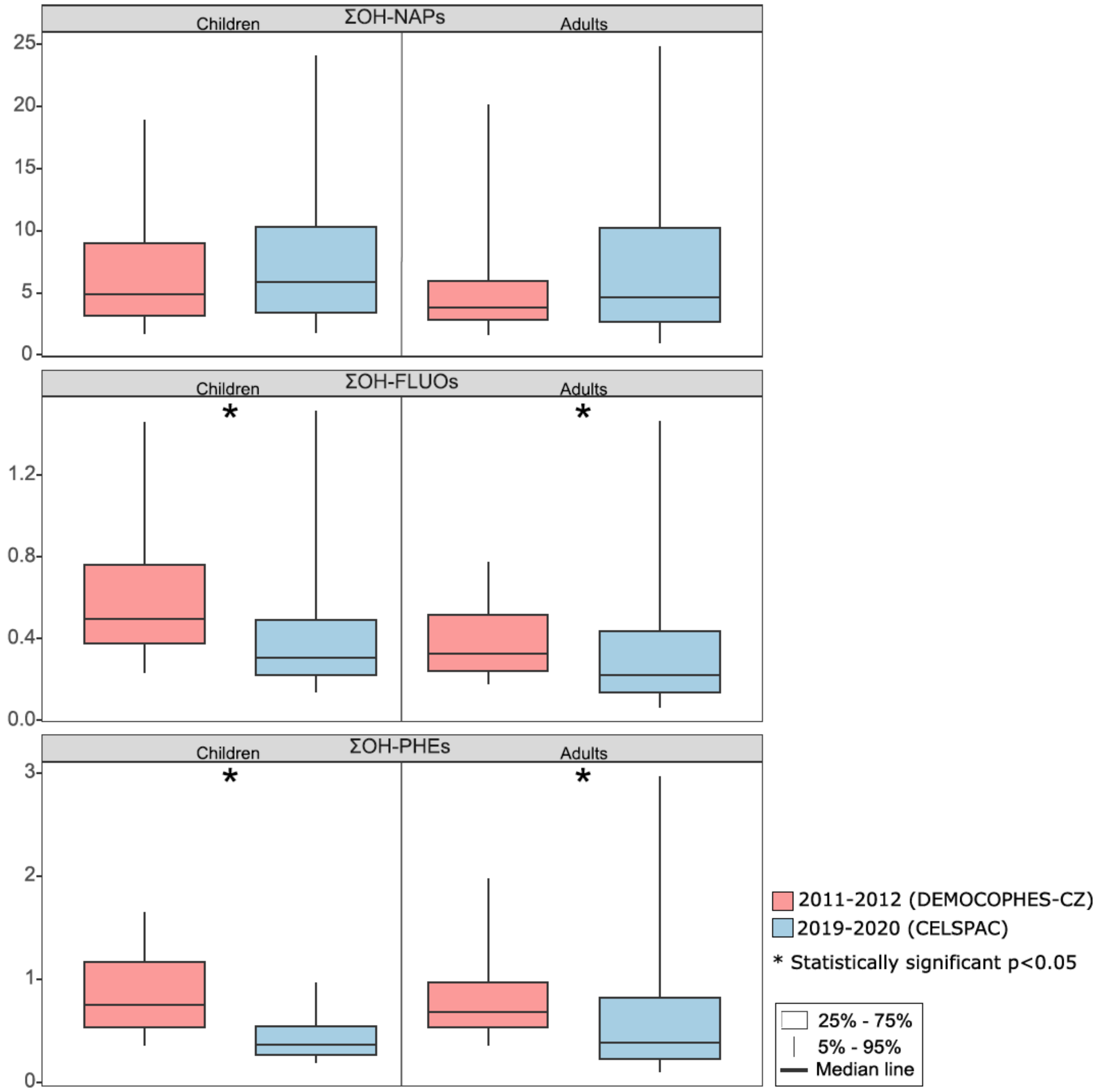

Figure S6 Comparison of the sums of OH-PAH metabolite concentrations (μg/g creatinine) by parent PAH between DEMOCOPHES-CZ and CELSPAC adult women, and between DEMOCOPHES-CZ and CELSPAC children in two sampling periods. Smokers were excluded.

Table S5 Regression analysis results (regression coefficients and standard error SE). The coefficient of determination R^2 is given for a model including only statistically significant factors.

	**OH-NAPs**			**1-OH-NAPs**			**2-OH-NAPs**			**OH-FLUOs**			**OH-PHEs**			**1-OH-PYR**		
DEMOCOPHES-CZ MOTHERS																		
	β	SE	p	β	SE	p	β	SE	p	β	SE	p	β	SE	p	β	SE	p
smoking	0.58	0.09	0	0.67	0.07	<0.001	0.56	0.07	<0.001	0.64	0.09	0	0.38	0.11	<0.001	0.48	0.1	<0.001
fish consumption	0.3	0.13	0.03															
locality (city/towns)	-0.15	0.09	0.09				-0.16	0.07	0.05									
Adjusted R^2	0.43			0.45			0.35			0.45			0.09			0.26		
DEMOCOPHES-CZ CHILDREN																		
fish consumption	0.22	0.09	0.01	0.2	0.09	0.03	0.19	0.09	0.05									
locality (city/towns)	-0.22	0.09	0.01	-0.21	0.09	0.02												
sex (female)													0.25	0.09	0.01	0.23	0.09	0.01
Adjusted R^2	0.08			0.07			0.04			ns			ns			ns		
CELSPAC CHILDREN																		
Season (cold)	0.15	0.07	0.05				0.15	0.08	0.09				0.13	0.07	0.05			
Locality (rural)	-0.21	0.08	0.02	-0.18	0.08	0.03	-0.21	0.08	0.02	-0.17	0.07	0.03	-0.2	0.08	0.02	-0.22	0.75	0.01
Cooking (electricity)													-0.23	0.1	0.02			
Adjusted R^2	0.04			ns			0.03			0.06			0.07			0.05		
CELSPAC TEENAGERS																		
age													0.12	0.06	0.05			
offal													0.16	0.07	0.01			
Cooking (Electricity)																-0.16	0.8	0.05
Adjusted R^2	ns			ns			ns			ns			0.04			ns		

Mother smoking - number of cigarettes per day, fish consumption -frequency of fish consumption (including all sea product) per week, locality- type of locality (city x rural area), factory near -factory within a 50 m radius around house.

Table S6 Pairwise comparison of OH-PAH metabolite concentrations for mothers and children from the DEMOCOPHES-CZ study, N=116 mother-children's pairs

metabolite	Median mothers	Median children	Spearman's correlation children x mother	p-value WM Pairs Test
OH-NAPs	4.85	4.63	0.34	0.051
OH-FLUOs	0.49	0.36	0.46	0.000
OH-PHEs	0.74	0.70	0.45	0.980
1-OH-PYR	0.12	0.08	0.44	0.000

Table S7 Descriptive statistics of the creatinine adjusted urinary concentrations of PAH metabolites in smoking and non-smoking mothers from the DEMOCOPHES-CZ. Values below LOD were replaced by LOD/√2.

Cohort study	Biomarker	Creatinine adjusted (µg/g creatinine)							
		Min	5P	25P	Median	GM	75P	95P (95% CI)	Max
NON-SMOKING MOTHERS (DEMOCOPHES-CZ), N=92	1-OH-NAP	<LOD	0.690	1.094	1.510	1.790	2.938	5.941	67.979
	2-OH-NAP	<LOD	0.841	1.538	2.254	2.364	3.415	8.740	28.933
	2-OH-FLUO	0.108	0.136	0.208	0.269	0.296	0.413	0.675	2.171
	3-OH-FLUO	<LOD	<LOD	0.030	0.051	0.054	0.091	0.167	0.235
	1-OH-PHE	0.078	0.113	0.189	0.270	0.275	0.367	0.705	1.130
	2-3-OH-PHE	<LOD	<LOD	0.153	0.211	0.231	0.323	0.716	0.912
	4-OH-PHE	<LOD	<LOD	0.039	0.055	0.061	0.089	0.197	0.386
	9-OH-PHE	<LOD	<LOD	0.096	0.152	0.142	0.205	0.352	3.281
	1-OH-PYR	<LOD	<LOD	<LOD	0.072	0.079	0.116	0.199	0.400
	3-OH-BaP	<LOD	<LOD	<LOD	<LOD	<LOD	<LOD	0.168	0.229
	ΣOH-NAP	1.192	1.547	2.771	3.784	4.420	6.061	20.057	72.341
	ΣOH-FLUO	0.121	0.176	0.238	0.325	0.353	0.525	0.776	2.360
	ΣOH-PHE	0.207	0.357	0.530	0.679	0.751	0.978	1.968	4.482
SMOKING MOTHERS (DEMOCOPHES-CZ), N=24	1-OH-NAP	1.056	1.297	3.394	6.790	6.967	18.760	29.778	42.334
	2-OH-NAP	1.733	1.847	3.045	5.364	6.298	12.782	20.520	30.054
	2-OH-FLUO	0.216	0.233	0.372	0.718	0.750	1.456	2.643	3.089
	3-OH-FLUO	0.039	0.041	0.084	0.181	0.210	0.528	0.841	1.085
	1-OH-PHE	<LOD	0.061	0.209	0.321	0.308	0.563	0.787	1.686
	2-3-OH-PHE	0.118	0.131	0.179	0.309	0.283	0.377	0.566	0.662
	4-OH-PHE	<LOD	0.011	0.037	0.068	0.060	0.104	0.161	0.227
	9-OH-PHE	<LOD	<LOD	0.129	0.182	0.218	0.575	1.137	1.400
	1-OH-PYR	0.033	0.040	0.076	0.150	0.130	0.207	0.327	0.335
	3-OH-BaP	<LOD	<LOD	<LOD	<LOD	<LOD	<LOD	0.153	0.206
	ΣOH-NAP	2.998	3.133	7.259	12.380	13.665	33.126	48.368	72.389
	ΣOH-FLUO	0.255	0.279	0.456	0.918	0.970	2.165	3.484	4.174
	ΣOH-PHE	0.267	0.291	0.614	0.996	0.964	1.675	2.413	3.229

Table S8 Descriptive statistics of the volume based urinary concentrations of PAH metabolites (µg/L) for smoking and non-smoking mothers from the DEMOCOPHES-CZ. Values below LOD were replaced by LOD/√2.

Cohort study	Biomarker	Volume based (µg/L)							
		Min	5P	25P	Median	GM	75P	95P	Max
NON-SMOKING MOTHERS (DEMOCOPHES-CZ), N=92	1-OH-NAP	<LOD	0.347	1.073	1.618	1.814	2.977	9.093	42.157
	2-OH-NAP	<LOD	0.512	1.380	2.433	2.395	3.932	9.018	82.297
	2-OH-FLUO	0.042	0.105	0.192	0.306	0.300	0.441	1.028	1.606
	3-OH-FLUO	<LOD	<LOD	0.034	0.052	0.055	0.095	0.199	0.328
	1-OH-PHE	0.032	0.082	0.164	0.291	0.279	0.432	0.948	1.370
	2-3-OH-PHE	<LOD	<LOD	0.135	0.237	0.234	0.390	0.781	1.624
	4-OH-PHE	<LOD	<LOD	0.035	0.058	0.061	0.121	0.257	0.345
	9-OH-PHE	<LOD	<LOD	0.097	0.149	0.144	0.204	0.476	4.520
	1-OH-PYR	<LOD	<LOD	<LOD	0.084	0.080	0.138	0.225	0.579
	3-OH-BaP	<LOD	<LOD	<LOD	<LOD	<LOD	<LOD	0.097	0.198
	ΣOH-NAP	0.474	0.893	2.535	4.235	4.478	7.297	17.569	98.973
	ΣOH-FLUO	0.056	0.119	0.236	0.356	0.358	0.518	1.166	1.893
	ΣOH-PHE	0.138	0.226	0.490	0.732	0.761	1.104	2.552	6.175
SMOKING MOTHERS (DEMOCOPHES-CZ), N=24	1-OH-NAP	0.611	0.930	4.464	7.493	7.826	18.541	36.730	39.957
	2-OH-NAP	0.700	1.447	4.188	7.073	7.074	16.411	27.781	28.367
	2-OH-FLUO	0.130	0.209	0.465	0.786	0.843	1.947	2.930	3.208
	3-OH-FLUO	0.026	0.039	0.101	0.208	0.235	0.641	1.028	1.106
	1-OH-PHE	<LOD	0.064	0.242	0.398	0.346	0.579	1.187	1.452
	2-3-OH-PHE	0.128	0.140	0.201	0.318	0.318	0.439	0.783	0.988
	4-OH-PHE	<LOD	0.014	0.044	0.066	0.067	0.108	0.284	0.333
	9-OH-PHE	<LOD	<LOD	0.123	0.212	0.245	0.539	1.206	1.778
	1-OH-PYR	0.059	0.061	0.079	0.155	0.146	0.253	0.441	0.707
	3-OH-BaP	<LOD	<LOD	<LOD	<LOD	<LOD	<LOD	0.141	0.150
	ΣOH-NAP	1.311	2.449	9.798	15.177	15.348	34.261	64.512	68.323
	ΣOH-FLUO	0.156	0.248	0.584	0.995	1.090	2.500	3.941	3.973
	ΣOH-PHE	0.336	0.372	0.699	1.135	1.083	1.793	2.552	3.949

Table S9 Descriptive statistics of the urinary concentrations of PAH metabolites in CELSPAC cohort (children, teenagers and young adults summarized together) using either creatinine, specific gravity or no standardization.

Adjustment	Analyte	Summary statistic							
		Min	5P	25P	Median	GM	75P	95P	Max

Volume based concentrations (µg/L) N=809	1-OH-NAP	<LOD	0.300	0.790	1.540	1.655	3.203	11.115	63.800
	2-OH-NAP	<LOD	0.960	2.338	4.230	4.386	8.475	20.505	114.000
	2-OH-FLUO	<LOD	0.080	0.160	0.270	0.284	0.480	1.390	24.400
	3-OH-FLUO	<LOD	0.014	0.030	0.060	0.065	0.120	0.420	3.900
	1-OH-PHE	<LOD	0.021	0.030	0.050	0.061	0.110	0.380	7.260
	2-3-OH-PHE	<LOD	0.049	0.110	0.170	0.185	0.310	0.690	11.800
	4-OH-PHE	<LOD	0.014	0.020	0.060	0.055	0.110	0.280	2.960
	9-OH-PHE	<LOD	0.042	0.042	0.042	0.098	0.290	0.661	23.000
	1-OH-PYR	<LOD	0.035	0.060	0.100	0.099	0.160	0.340	11.900
	3-OH-BaP	<LOD	0.057	0.057	0.057	0.057	0.057	0.057	3.580
	ΣOH-NAP	0.474	1.400	3.488	6.020	6.353	12.063	31.801	139.200
	ΣOH-FLUO	0.035	0.094	0.190	0.340	0.357	0.600	1.731	28.300
	ΣOH-PHE	0.127	0.143	0.237	0.427	0.464	0.830	1.880	45.020
Creatinine-adjusted concentrations (µg/g creat) N=809	1-OH-NAP	0.059	0.217	0.603	1.165	1.238	2.314	8.784	232.552
	2-OH-NAP	0.125	0.770	1.752	3.183	3.279	6.333	15.356	159.532
	2-OH-FLUO	0.010	0.050	0.125	0.190	0.213	0.340	1.001	17.223
	3-OH-FLUO	0.002	0.010	0.025	0.043	0.048	0.084	0.312	7.381
	1-OH-PHE	0.005	0.010	0.021	0.040	0.045	0.094	0.287	3.943
	2-3-OH-PHE	0.014	0.042	0.085	0.129	0.140	0.219	0.549	12.977
	4-OH-PHE	0.003	0.009	0.020	0.040	0.041	0.074	0.247	8.016
	9-OH-PHE	0.010	0.018	0.031	0.053	0.073	0.156	0.498	12.491
	1-OH-PYR	0.009	0.023	0.045	0.070	0.074	0.111	0.266	6.463
	3-OH-BaP	0.010	0.019	0.029	0.041	0.043	0.058	0.104	1.029
	ΣOH-NAP	0.226	1.070	2.526	4.682	4.749	8.932	24.424	392.085
	ΣOH-FLUO	0.017	0.071	0.154	0.235	0.267	0.437	1.409	24.605
	ΣOH-PHE	0.035	0.106	0.206	0.310	0.347	0.540	1.555	30.041
Specific gravity adjusted concentration (µg/L) N=809	1-OH-NAP	0.177	0.449	1.081	2.000	2.152	3.779	14.063	305.739
	2-OH-NAP	0.242	1.510	3.023	5.529	5.702	10.212	26.254	209.739
	2-OH-FLUO	0.023	0.106	0.211	0.347	0.370	0.581	1.883	28.566
	3-OH-FLUO	0.009	0.019	0.043	0.076	0.084	0.144	0.485	9.704
	1-OH-PHE	0.015	0.022	0.037	0.069	0.079	0.146	0.455	8.500
	2-3-OH-PHE	0.037	0.078	0.146	0.210	0.241	0.373	0.875	17.061
	4-OH-PHE	0.010	0.015	0.033	0.071	0.072	0.128	0.431	10.539
	9-OH-PHE	0.028	0.037	0.051	0.081	0.128	0.291	0.940	26.927
	1-OH-PYR	0.025	0.045	0.081	0.120	0.129	0.186	0.430	13.932
	3-OH-BaP	0.036	0.044	0.054	0.068	0.074	0.093	0.162	2.644
	ΣOH-NAP	0.503	2.028	4.423	7.844	8.258	14.501	39.278	515.478
	ΣOH-FLUO	0.038	0.134	0.258	0.418	0.465	0.723	2.353	33.132
	ΣOH-PHE	0.096	0.199	0.341	0.531	0.603	0.948	2.505	52.706

Table S10 Setting up filters to search for specific PAHs and sites on Global Environmental Assessment and Information System (GENASIS). On the website https://data.genasis.cz/#/outdoor/spatial-distribution it has to be selected the "*Time Series Analysis*" option and enter the searching filters correctly.

**Locality of monitoring**	**Filters to be set**				
	Material	Site	Parameter	Unit	Project
Košetice	Outdoor Air Passive PUF	Kosetice, EMEP station	naphthalene / phenanthrene / fluorene*	ng sample-1	MONET – CZ
Brno (Líšeň)	Outdoor Air Passive PUF	Brno, Lisen, CHMI station	naphthalene / phenanthrene / fluorene*	ng sample-1	MONET – CZ
Praha (Libuš)	Outdoor Air Passive PUF	Praha, Libus, EMEP	naphthalene / phenanthrene / fluorene*	ng sample-1	MONET – CZ
Liberec (Ještěd)	Outdoor Air Passive PUF	Liberec, Jested	naphthalene / phenanthrene / fluorene*	ng sample-1	MONET – CZ

* select always just one PAH

Table S11 Median concentrations (ug/L, no adjusted) of OH-PAH measured in various cohorts in the world.

Area	Country	Cohort	Age	Sampling year	N	Reference	1-NAP	2-NAP	2-FLU	3-FLU	1-PHE	2-PHE	3-PHE	4- PHE	9-PHE	1-PYR
Europe	DEMOCOPH ES-CZ	CH	6-11	2011-12	199	our study	1.93	2.7	0.402	0.102	0.267	0.254 [a]		0.053	0.122	0.131
		A: Females	28-47	2011-12	116	our study	2.05	2.82	0.349	0.065	0.321	0.257 [a]		0.061	0.156	0.095
	CZ-CELSPAC	CH	9-11	2019-20	195	our study	2	4.33	0.31	0.06	0.14	0.16 [a]		0.03	<LOD	0.09
		TE	12-17	2019-20	299	our study	1.52	4.27	0.25	0.07	0.04	0.17 [a]		0.07	<LOD	0.1
		YA	18-37	2019-20	315	our study	1.27	4.13	0.27	0.05	0.04	0.18 [a]		0.09	0.21	0.09
	CZE	A: Pregnant Females	18-43	2016-17	330	(12)	0.36	4.66	0.23							0.12
	BE FLEHS	TE	14–15	2017-18	412-414	(13)		3.5	0.175 [b]		0.093 [c]	0.066	0.065	<LOQ	0.093 [c]	0.06
	DE GerES V	CH and TE	3-17	2014-17	516	(14)	0.74	4.08	0.45		0.14	0.08	0.13	0.04	0.06	0.1
		CH	6-10	2014–17	166		0.76	3.6	0.44		0.15	0.07	0.13	0.04	0.06	0.09
		CH	11-13	2014–17	103		0.66	3.42	0.39		0.13	0.09	0.14	0.04	0.06	0.11
		TE	14-17	2014–17	149		0.71	5.44	0.51		0.15	0.09	0.12	0.05	0.06	0.13
	DE	YA: NS	20-28	2017	57	(15)	0.29	0.72			0.12	0.04	0.06	0.02	0.07	0.05
		YA: NS	20-29	2018	59		0.23	0.87			0.1	0.04	0.05	0.01	0.04	0.04
		YA: NS	20-28	2019	54		0.19	0.94			0.09	0.03	0.05	0.01	0.05	0.04
	ES	TE + A	16–65	2009–10	957	(16)							0.2 [d]			0.18
	SE [e]	Males, all	20-63	2010-11, 2015	130-152	(17)						0.14				0.06
	HBM4EU [f]	YA	20-39	2014-21	1031-2601	(18)	0.637	4.45	0.258	0.054	0.105	0.063	0.09	0.028		0.097
North	CA CHMS	CH	6-11	2014-15	494-510	(19)	0.57	3.6	0.22	0.082	0.12	0.04	0.083	0.017	0.026	0.097
		TE	12-19	2014-15	498		0.65	4.8	0.26	0.089	0.15	0.15	0.059	0.091	0.028	0.01
		YA	20-39	2014-15	352		0.97	5.4	0.32	0.1	0.17	0.077	0.099	0.03	0.045	0.12

	USA US NHANES	CH	6-11	2015-16	373-375	(20)	0.835	3.98	0.123	0.058	0.077	0.096 [a]				0.121
		TE	12-19	2015-16	392-398		0.87	4.95	0.149	0.072	0.094	0.117 [a]				0.12
		A	20+	2015-16	1510-1790		1.76	4.96	0.205	0.0902	0.109	0.126 [a]				0.122
Australia	AUS [f]	CH	5-14	2012-13	400	(6)	1.52	2.95	0.156	0.071	0.111	0.05	0.07	0.017		0.134
		ALL	0-60+	2012-13	300		9.2	4.1	0.261	0.132	0.136	0.06	0.081	0.03		0.142
Asia	KOR KoNEHS	A	19+	2012-14	6397–6418	(21)		2.07	0.26		0.09					0.15
		A: Males	19+	2012-15	2735–2750			3.1	0.39		0.11					0.18
		A: Females	19+	2012-16	3660–3673			1.56	0.18		0.08					0.13
	IRN	CH + TE	6-18	2015–16	150	(22)	0.36	0.424							0.107	0.099
	ISR	A	20-74	2011	250	(23)					0.219	0.095	0.141	0.032		0.204
	SAU	CH: Boys	10-12	2013	170	(24)					0.157		0.156	0.035		0.221
	CHN	CH	7-14	2015	1206	(25)		2.9	0.971						3.18	0.224
	CHN	A	30-51	2010-12	84	(26)	0.528	2.27	0.893		0.323	0.032	0.0387	0.033		0.378
	IND	A	30-51	2010-12	38		1.11	3.78	0.346		0.256	0.256	0.289	0.027		0.424
	JPN	A	30-51	2010-12	34		0.266	3.25	0.207		0.065	0.065	0.121	0.01		0.075
	KWT	A	30-51	2010-12	38		1.41	7.33	0.448		0.089	0.089	0.137			0.22
	MYS	A	30-51	2010-12	29		0.263	1.55	0.112		0.036	0.036	0.043	0.006		0.065
	VNM	A	30-51	2010-12	23		0.642	4.9	0.473		0.157	0.157	0.2	0.032		0.463
	VNM	A	30-51	2010-12	23		0.642	4.9	0.473		0.157	0.157	0.2	0.032		0.463

[a]2+3-PHE, [b]2+3-FLU, [c]1+9-PHE, [d]1+2+3+4+9-PHE, [e]adjusted concentration (µg/g creatinine), [f]geometric mean

1. Esteban López M, Göen T, Mol H, Nübler S, Haji-Abbas-Zarrabi K, Koch HM, et al. The European human biomonitoring platform - Design and implementation of a laboratory quality assurance/quality control (QA/QC) programme for selected priority chemicals. Int J Hyg Environ Health. 2021 May;234:113740.
2. Nübler S, Esteban López M, Castano A, Mol H, Müller J, Schäfer M, et al. External Quality Assurance Schemes (Equass) and Inter-Laboratory Comparison Investigations (Icis) for Human Biomonitoring of Polycyclic Aromatic Hydrocarbon (Pah) Biomarkers in Urine as Part of the Quality Assurance Programme Under Hbm4eu. SSRN Electronic Journal. 2022;11(9):1092–9.
3. Dereziński P, Klupczyńska A, Sawicki W, Kokot ZJ. Creatinine determination in urine by liquid chromatography-electrospray ionization-tandem mass spectrometry method. Acta Poloniae Pharmaceutica - Drug Research. 2016;73(2):303–13.
4. Falcó G, Domingo JL, Llobet JM, Teixidó A, Casas C, Müller L. Polycyclic Aromatic Hydrocarbons in Foods: Human Exposure through the Diet in Catalonia, Spain. J Food Prot. 2003;66(12):2325–31.
5. Perera FP, Viswanathan S, Whyatt R, Tang D, Miller RL, Rauh V. Children’s environmental health research - Highlights from the Columbia Center for Children’s Environmental Health. Ann N Y Acad Sci. 2006;1076:15–28.
6. Thai PK, Heffernan AL, Toms LML, Li Z, Calafat AM, Hobson P, et al. Monitoring exposure to polycyclic aromatic hydrocarbons in an Australian population using pooled urine samples. Environ Int. 2016 Mar 1;88:30–5.
7. Jung SK, Choi W, Kim SY, Hong S, Jeon HL, Joo Y, et al. Profile of Environmental Chemicals in the Korean Population—Results of the Korean National Environmental Health Survey (KoNEHS) Cycle 3, 2015–2017. Int J Environ Res Public Health. 2022;19(2).
8. Huang X, Deng X, Li W, Liu S, Chen Y, Yang B, et al. Internal exposure levels of polycyclic aromatic hydrocarbons in children and adolescents: A systematic review and meta-analysis. Environ Health Prev Med. 2019;24(1):1–15.
9. Li Z, Sandau CD, Romanoff LC, Caudill SP, Sjodin A, Needham LL, et al. Concentration and profile of 22 urinary polycyclic aromatic hydrocarbon metabolites in the US population. 2008;107:320–31.
10. Barr DB, Wilder LC, Caudill SP, Gonzalez AJ, Needham LL, Pirkle JL. Urinary creatinine concentrations in the U.S. population: Implications for urinary biologic monitoring measurements. Environ Health Perspect. 2005;113(2):192–200.

11. Borůvková J. GJ, ŠK, BZ, KJ, HR, DL, HI, KJ. http://www.genasis.cz. 2015. GENASIS – Global Environmental Assessment and Information System.

12. Urbancova K, Dvorakova D, Gramblicka T, Sram RJ, Hajslova J, Pulkrabova J. Comparison of polycyclic aromatic hydrocarbon metabolite concentrations in urine of mothers and their newborns. Science of The Total Environment. 2020 Jun 25;723:138116.

13. Schoeters G, Verheyen VJ, Colles A, Remy S, Martin LR, Govarts E, et al. Internal exposure of Flemish teenagers to environmental pollutants: Results of the Flemish Environment and Health Study 2016–2020 (FLEHS IV). Int J Hyg Environ Health. 2022;242(April):113972.

14. Murawski A, Roth A, Schwedler G, Schmied-Tobies MIH, Rucic E, Pluym N, et al. Polycyclic aromatic hydrocarbons (PAH) in urine of children and adolescents in Germany – human biomonitoring results of the German Environmental Survey 2014–2017 (GerES V). Int J Hyg Environ Health. 2020;226(February):113491.

15. Burkhardt T, Scherer M, Scherer G, Pluym N, Weber T, Kolossa-Gehring M. Time trend of exposure to secondhand tobacco smoke and polycyclic aromatic hydrocarbons between 1995 and 2019 in Germany – Showcases for successful European legislation. Environ Res. 2023;216(October 2022).

16. Bartolomé M, Ramos JJ, Cutanda F, Huetos O, Esteban M, Ruiz-Moraga M, et al. Urinary polycyclic aromatic hydrocarbon metabolites levels in a representative sample of the Spanish adult population: The BIOAMBIENT.ES project. Chemosphere. 2015;135:436–46.

17. Alhamdow A, Lindh C, Albin M, Gustavsson P, Tinnerberg H, Broberg K. Early markers of cardiovascular disease are associated with occupational exposure to polycyclic aromatic hydrocarbons. Sci Rep. 2017;7(1):1–11.

18. Govarts E, Gilles L, Rodriguez Martin L, Santonen T, Apel P, Alvito P, et al. Harmonized human biomonitoring in European children, teenagers and adults: EU-wide exposure data of 11 chemical substance groups from the HBM4EU Aligned Studies (2014–2021). Vol. 249, International Journal of Hygiene and Environmental Health. Elsevier GmbH; 2023. p. 114119.

19. Health Canada. Fourth Report on Human Biomonitoring of Environmental Chemicals in Canada. 2017.

20. CDC. National Report on Human Exposure to Environmental Chemicals. 2020.

21. Choi W, Kim S, Baek YW, Choi K, Lee K, Kim S, et al. Exposure to environmental chemicals among Korean adults-updates from the second Korean National

Environmental Health Survey (2012–2014). Int J Hyg Environ Health. 2017;220(2):29–35.

22. Kelishadi R, Sobhani P, Poursafa P, Amin MM, Ebrahimpour K, Hovsepian S, et al. Is there any association between urinary metabolites of polycyclic aromatic hydrocarbons and thyroid hormone levels in children and adolescents? Environmental Science and Pollution Research. 2018;25(2):1962–8.

23. Levine H, Berman T, Goldsmith R, Göen T, Spungen J, Novack L, et al. Urinary concentrations of polycyclic aromatic hydrocarbons in Israeli adults: Demographic and life-style predictors. Int J Hyg Environ Health. 2015;218(1):123–31.

24. Alghamdi MA, Alam MS, Stark C, Mohammed N, Harrison RM, Shamy M, et al. Urinary metabolites of polycyclic aromatic hydrocarbons in Saudi Arabian schoolchildren in relation to sources of exposure. Environ Res. 2015;140:495–501.

25. Liu S, Liu Q, Ostbye T, Story M, Deng X, Chen Y, et al. Levels and risk factors for urinary metabolites of polycyclic aromatic hydrocarbons in children living in Chongqing, China. Science of the Total Environment. 2017;598(1):553–61.

26. Guo Y, Senthilkumar K, Alomirah H, Moon HB, Minh TB, Mohd MA, et al. Concentrations and profiles of urinary polycyclic aromatic hydrocarbon metabolites (OH-PAHs) in several Asian countries. Environ Sci Technol. 2013;47(6):2932–8.
